# Plumage and Fat Condition Scores as Well-Being Assessment Indicators in a Small Passerine Bird, the Zebra Finch (*Taeniopygia guttata*)

**DOI:** 10.3389/fvets.2022.791412

**Published:** 2022-04-14

**Authors:** Lisa Kalnins, Oliver Krüger, E. Tobias Krause

**Affiliations:** ^1^Department of Animal Behaviour, Bielefeld University, Bielefeld, Germany; ^2^Institute of Animal Welfare and Animal Husbandry, Friedrich-Loeffler-Institut, Celle, Germany

**Keywords:** animal welfare, Zebra Finch, songbird, plumage score, fat score, observer reliability, feather condition

## Abstract

The well-being of animals kept by humans in laboratories, farms, or as pets should always be of the highest importance, and a prerequisite for this is adequate housing. To assess the potential indicators for animal well-being, ideally simple, non-invasive, and reliable methods are necessary. Here, we propose a novel plumage scoring system for small songbirds, using the example of the Zebra Finch, and examine its reliability in comparison with a well-known body condition index, that is, the fat score. We used up to five different observers of different experience levels to assess inter- and intra-observer reliability of the proposed plumage score and also the fat score. We found substantial inter-observer reliability for the proposed novel plumage score, and lower inter-observer reliability for the fat score, which seems to require more training of observers. The intra-observer reliability of the experienced observer who trained the others also showed a very strong reliability for the plumage score and for the fat score. Thus, we conclude that our proposed novel plumage score is a simple, reliable, and non-invasive way to estimate an important indicator of captive Zebra Finches' well-being. Furthermore, the plumage score can be reliably taught to other observers. The plumage score, maybe in combination with the fat score, may be an important tool to reliably assess well-being on a regular basis in captive populations in zoos, laboratories, or pet stocks.

## Introduction

Assessing the well-being of captive birds should be mandatory for any animal owner. There are several approaches to assess the indicators of health condition and thus indicators of well-being in birds. Specifically in poultry, several approaches are commonly used to assess the well-being indicators on farms. For poultry, especially fattening poultry such as broiler chickens or turkeys, locomotor behavior is often used for assessing well-being (1–4). Locomotion is more often impaired than feather status due to the rapid growth of these birds. Therefore, locomotor assessment is more suited in fattening poultry. For laying hens, such as domestic chicken or quails, well-being assessment indicators are mainly based on the phenotypic plumage parameters (5, 6), as they provide robust proxies for animal condition.

However, assessing well-being indicators in birds is not only important in farmed animals, but is also a key issue for avian species housed in research institutes, zoological gardens, or as pets. In these scenarios, passerines are often kept, for example, the Zebra Finch [*Taeniopygia guttata*, Buchanan and Griffith (7)]. For these birds, which are not bred for increased fattening or egg production, phenotypic traits for *ad hoc* well-being indicator assessment will be most suited, that is, plumage scores or direct body conditions indices. Scoring the plumage of a bird, based on the density or relative amount of surface being feathered, reveals the differences among individuals and populations which can be linked to age, housing, or feeding conditions and thus may be important and informative well-being indicators. Birds in good conditions and/or health usually have a proper plumage as they can perform respective maintenance behavior in their housing environment and can carry the energetic costs. On the other hand, damaged and incomplete plumage may indicate poorer condition and/or health as a result of poorer environments, or previous agonistic interactions with conspecifics (8). A standardized scoring method is the necessary basis to systematically answer questions about well-being and any underlying reasons related to the differences in plumage appearance, such as the prevalence of feather pecking (9, 10). The assessment of the overall status of the plumage has often been neglected in avian research model species such as Great Tits (*Parus major*) or Zebra Finches (7). Commonly, only the colouration of certain ornaments of the plumage or the development of these has been considered (11–13), since plumage ornaments are also used by the birds themselves to assess the conditions of a conspecific. Well-being indicators of Zebra Finches have also been assessed using different hormonal changes over time (14), which is a good proxy, but a much more invasive, time-consuming, and expensive approach as blood sampling is required. In total, well-being, health condition, and other indicators related to well-being have been systematically recorded far less often in these bird species.

Besides the plumage condition, the amount of visible subcutaneous fat is a common method to evaluate the energy reserves of birds and thus assess their condition and thus having an indicator for their health which is also important for well-being. This method is also more commonly used in smaller birds such as passerines and may be a well-suited addition to a feather score for passerines. Both methods combined may allow to obtain important well-being indicators with minimal handling. Fat scores as the indicators of condition and health are also widely used in both migratory and non-migratory birds (15–17). A correlation between fat score and actual body fat percentage has been shown repeatedly (18, 19), which may be important to assess body condition and if this is done properly, it is relevant for the well-being of birds. Housing of captive animals can affect fat reserves and body weight; hence, this method is of interest in assessing husbandry effects. Wild-caught animals may, for example, suffer from stress in captivity and lose weight (20) and environmental stress in poultry may reduce the food intake and thus impair weight gain and reduce fat reserves (21). In pet animals, on the other hand, obesity as a consequence of incorrect housing or inappropriate feeding by owners is also common (22).

In this study, we aimed to establish a detailed plumage scoring system for one of the most important avian model species, the passerine Zebra Finch, to allow a more objective and informative evaluation of *ad hoc* animal conditions, health and well-being within and between housing conditions and laboratories. In addition, we further include a fat scoring system (23), as the fat scores are fast and easy to estimate during plumage assessment, and provide additional important information about well-being and health. As the proposed scoring schemes depend on the observers, we also evaluated them with respect to intra- and inter-observer reliability.

## Methods

### A Categorical Plumage Score for Small Passerines Using the Zebra Finch as an Example

**Figure 1:**
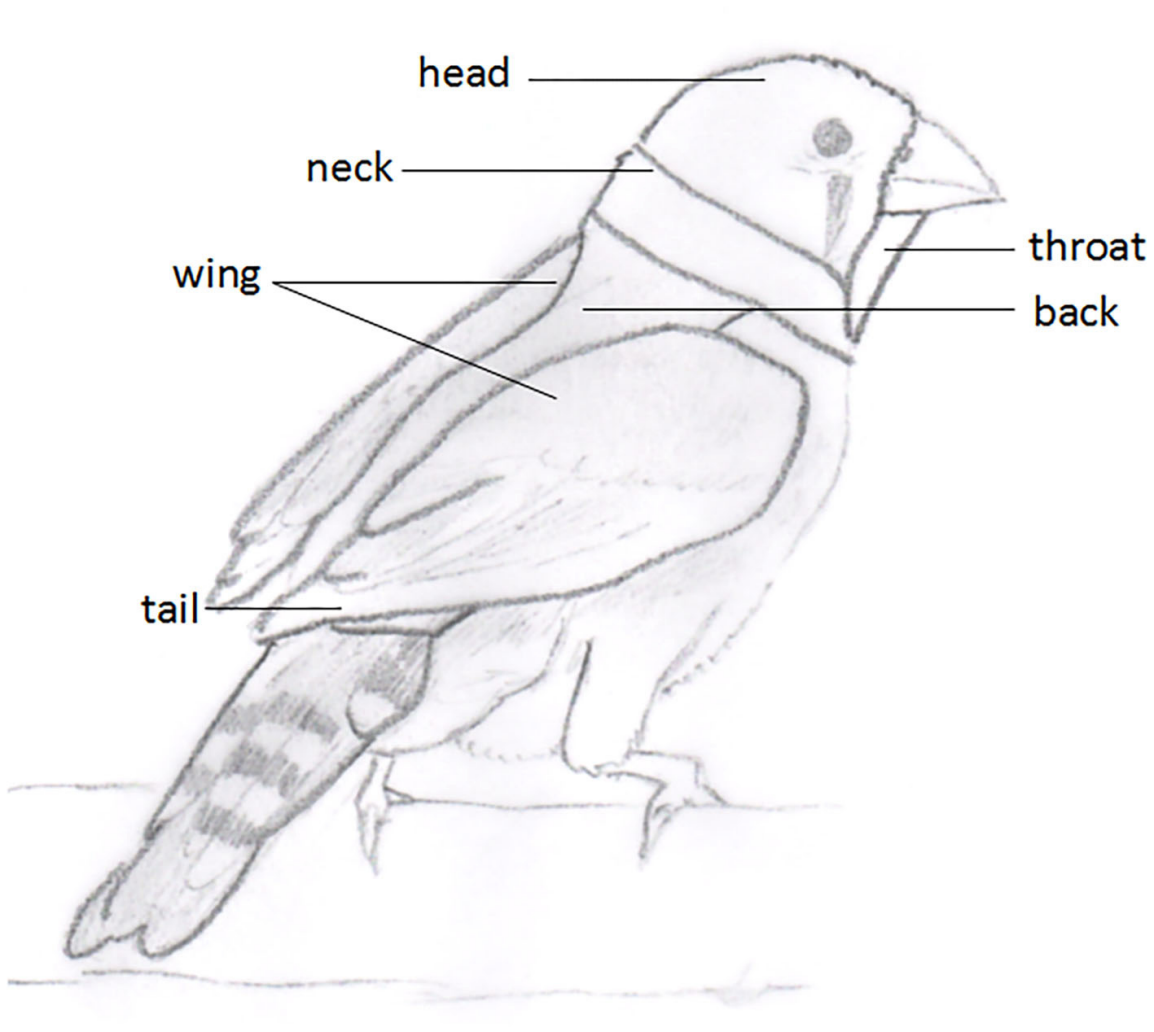
Schematic overview of the six plumage parts of the Zebra Finches that were considered in the plumage scoring system.

To develop a categorical plumage scoring system for Zebra Finches, we divided the plumage surface into several focal parts to get a detailed picture and to identify potential problem areas of individuals. Therefore, we used the following six plumage areas: i) head, ii) neck, iii) back, iv) wings, v) tail, and vi) throat (Figure 1), in line with similar areas used in poultry. The area “head” is defined as the feathered part of the bird's skull which is adjacent to the “neck”. Neck is defined as the feathered part around the flexible part of the connection between body and head. Between the underneath of the beak and the ventral section of the “neck” is the region “throat”. From the dorsal “neck”, between the bird's “wings”, down to the preen gland is the area defined as “back”. This is followed by the “tail”. The wings of the bird and, in particular, the flight feathers are the part of the area “wing.” The so-far mentioned regions represent the obvious visible regions of the plumage which can, in theory, also be recorded without handling birds. However, it is recommended and implemented here to gently take birds into the hand to estimate the plumage scores. Other plumage regions turned out to be ill-suited, at least in the Zebra Finch, for different reasons, as they are either hard to assess in practice and some regions vary with sex and reproductive status and may therefore reveal no reliable well-being-related information. Finally, the sides and legs were not included.

**Figure 2:**
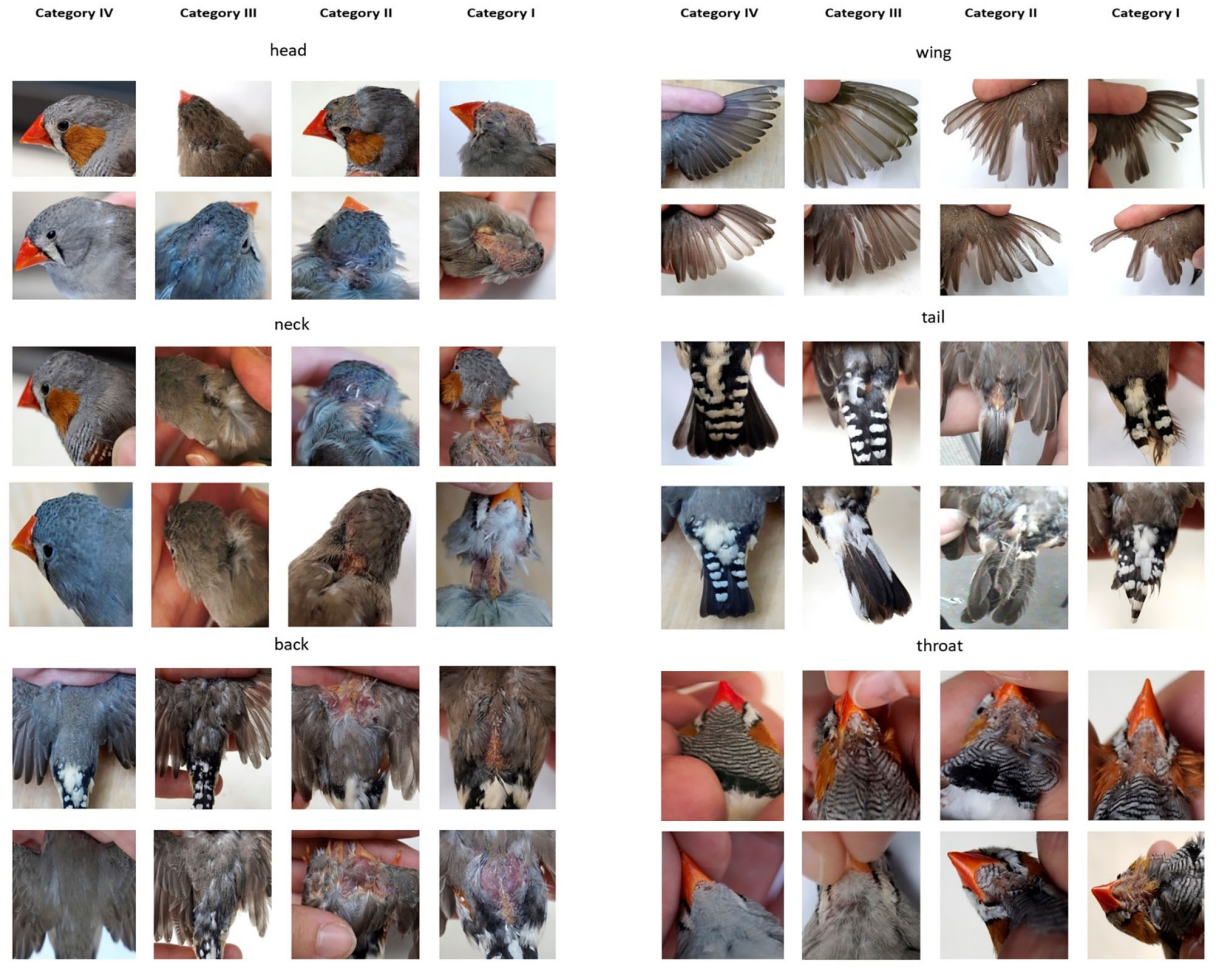
Systematic examples of the respective plumage scores for each scoring category at each of the six plumage regions of Zebra Finches.

At each of these six specified areas, plumage condition was categorized into one of four scores. The scoring categories were established in line with the procedures in laying hens (6). Thus, for each plumage area, one of four categorical values for that respective plumage status was assigned. Plumage categories were scored between I (worst) and IV (best). Category value IV is defined as a plumage in best condition, which means that the plumage is complete and dense, there are almost no significant gaps or damaged feathers (>95% undamaged, Figure 2), and flight feathers and tail feathers are complete and tidy. Category III means that the plumage is predominately complete with few gaps or damaged feathers. It can be fully feathered, but the feathers are tousled instead of smooth (>75% damaged). The flight feathers can show slight defects (category III, Figure 2). There are some of the smaller tail or wing feathers missing or the tails looks tousled. In category II, the number and dimension of gaps and damaged feathers increases (<75–>25% in undamaged status). For the body part “wing,” there are at least two flight feathers missing (category II, Figure 2), and for the body part “tail”, some of the long tail feathers are missing. Furthermore, a visible preen gland indicates a category II “tail”. The worst category is I. Here, the plumage contains big gaps, partly no feathers at all, highly damaged feathers, or even injured skin (<25% in undamaged status). At last, one wing shows a loss of one-third of its flight feathers (category I; Figure 2). A tail of the category I misses nearly all long tail feathers. By summing up the individual scores (head, neck, back, tail, wing, and throat), we get an overview of the bird's plumage condition. For each of the six body regions, exemplary pictures for each category are provided (Figure 2).

For the overall assessment of the plumage, we suggest a single value that allows a direct intuitive judgment of the feather condition, that is, the plumage damage score. To calculate the damage score of a bird, one subtracts the sum of the six bird's plumage scores from the maximum possible score (e.g., 24–21 = 3, the bird has a summed plumage score of 21 which means a damage score of 3). If required, each one of the six considered plumaged body regions can be analyzed separately. The lower the damage score, the better the bird's plumage. The above-mentioned scoring of the six different body regions can maximally result in a maximum of 24 score points (6 × category IV), or a minimum of 6 (6 × category I). Assuming feather scores of ≤ 2 at any single area indicates severe damage of the feathers, which means that the summed feather score of all six body regions cannot exceed 12 (6 × category II or lower); therefore, a damage score of 12 or higher is associated {[24–12 (or less) = 12 (or more)]} with severe feather damages. Similarly, an individual score > 3 and a total score of > 18–24 result in a damage score of 6 or lower [(24–18 (or more) = 6 (or less)] and would indicate very intact plumage.

### A Categorical Fat Score for Small Passerines Using the Zebra Finch as an Example

**Figure 3:**
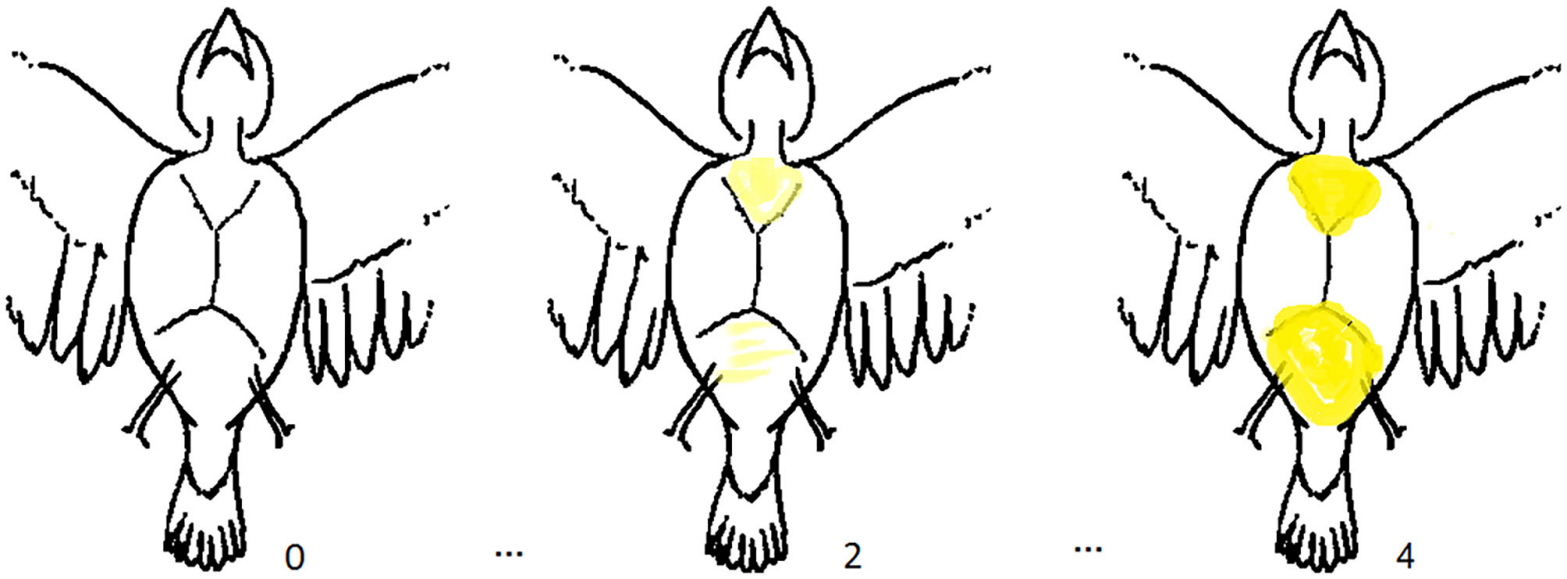
Outlined examples of the fat score.

In Zebra Finches, the subcutaneous fat stores are clearly visible and therefore quantifiable using appropriate scales. As Zebra Finches show a low amount of subcutaneous fat in general, we decided to adapt the score system used by Kaiser (23). We categorized the amount of visible fat on the ventral side of the birds (Figure 3), where especially the furcular and abdominal fat stores were of interest. In addition, we determined whether fat covers the wider abdominal and breast area. Birds with no visible fat in these focal areas received the score zero. Slight yellowish shades in the focal areas indicated a score of 1, clear yellow colouration indicated a score of 2 and slightly bulging fat deposited in one of the areas, or clear yellowish fat on both areas indicated a score of 3. Clear yellow colouration and a clear bulging of at least one area was scored as a 4. If the fat covered more areas of the abdomen and breast, this was scored as 5. The original score (23) included higher fat scores than 5. However, as the vast majority of Zebra Finches in our focal domesticated bird populations at Bielefeld University [referred to as “Bielefeld” in Forstmeier et al. (24); and “DOM Bielefeld” in Hoffman et al. (25)] are between 0 and a maximum of 4, we decided here against a further division above 5, which may, however, be implemented in other domesticated populations. Instead, as an addition to the originally suggested scoring scheme, we allowed a subdivision between two fat scores, that is, in 0.5 steps. This turned out to be very helpful, for example, if a bird shows more fat than for category 1, but not enough for category 2, thus we could assign a score of 1.5. This subdivision of the fat scores makes sense as one score reflects the entire body fat condition of the birds, while, for example, in the feather score, we assume that such a subdivision is not necessary, although easily possible, as there are already six different plumage scores, this is more subtle and reflects the bird's entire plumage condition.

Standardized and careful handling of the birds is important to generate reliable scores. The bird has to be held in one hand on its back. The neck is gently fixated between two fingers and the head is fixated carefully. The legs are held with the other hand. By gently puffing into the plumage, one can get a view of the bird's skin and the yellowish subcutaneous fat beneath.

### Reliability Assessment of the Examined Scorings

To assess the reliability of our proposed scoring system, we conducted reliability measures at two levels, that is, the inter-observer reliability and the intra-observer reliability.

### Inter-Observer Reliability

In total, 50 individual Zebra Finches were scored for the inter-observer reliability. About half of them were scored by 5 observers, and all of them by the two most experienced ones. To assess inter-observer reliability, 27 Zebra Finches (13 males, 14 females) were scored by five different observers independently within 10 min per bird. This short period was chosen to keep handling short for birds and to exclude differences in the bird's phenotype over longer periods such as days, weeks, or months. Observer #1 (L.K.) was most experienced, observer #2 was familiar with the scoring scheme, and the other tree observers #3–#5 were initially untrained with regard to the scoring scheme but received a short introduction of about 15 min from observer #1 in advance. However, all observers were very experienced with the general careful handling of Zebra Finches. The visual guide of the plumage scoring system (Figure 2) was available during data collection to all observers and also the illustrations of the fat scores. Additionally, the bird's body weight was taken after scoring to the nearest ± 0.01 g, using a digital balance (Kern EMB 600-2). Another 23 birds (2 females, 21 males), thus in sum *N* = 50 (16 females, 34 males), were scored by the observers #1 and #2 to assess the reliability with a larger sample size between the two most experienced observers.

For the reliability analysis, we did not calculate total plumage damage score, that is, the sum of all six plumage scores, but the six single scores of all six body parts were used, as this is a more conservative approach, which we assumed the best for the reliability analysis. This ensures that one can see exactly where the observers have scored different values, and subtle disagreements are not summed out (for further details refer to electronic Supplementary Table S1).

Statistical analyses were conducted using R (R version 4.1.0) (26). We analyzed the agreement of each possible observer pair with Spearman's rank correlation coefficients. Spearman's rank correlation coefficients r between 0 and 0.19 indicate a very week correlation, between 0.2 and 0.39 a weak correlation, between 0.4 and 0.69 a moderate correlation, between 0.7 and 0.89 a strong correlation, and between 0.9 and 1 a very strong correlation.

### Intra-Observer Reliability

To test the intra-observer reliability, observer #1 (L.K.) scored 22 birds (10 males, 12 females) five times within 1 day, that is, 7 h. Between each scoring was at least a break of 60 min for each bird to calm down and to eat and drink. The time gap was also important to reduce potential memory from the previous scores from the observer's mind, which of course was not fully possible, but at each recording event, 132 measures (6 body regions in 22 birds) were taken, so that the chance of remembering single scores was quite moderate. Birds were assessed as randomly as possible between sessions. The intra-observer analysis was conducted similarly to the above-described inter-observer reliability using Spearman's rank correlation coefficients for all pairs of the five measurements. Graphs for the agreements were made using the R-package “hexbin” (27).

### Ethical Note

All birds were taken from the stock of and remained at the Department of Animal Behavior, Bielefeld University. The measures were taken to assess and to establish a reliable well-being assessment measure. All persons handling the birds were, in general, experienced with the gentle and careful handling of the birds. Score collection was carried out in accordance with the German laws and required no specific permit as the brief handling was estimated to not stress the animals equivalent to, or higher than, that caused by the introduction of a needle in the skin in accordance with good veterinary practice (Directive 2010/63/EU). Breeding and housing of the birds were done with the permission of the local authorities, the Veterinäramt Bielefeld (no. 530.421630-1, 18.4.2002, and no. 530.4, 27.07.2014). All birds always had *ad libitum* food and water available and were housed in groups of at least 4 birds in cages or small aviaries. All birds were monitored daily.

## Results

### Inter-Observer Reliability

Our proposed plumage scores for Zebra Finches showed a substantial inter-observer reliability for the most experienced observer #1 in comparison with all four other observers (all *r* > 0.62, Table 1a). Considering reliability between all possible observer pairs, the Spearman's correlation coefficient was at least *r* > 0.56 (Table 1a). The average r-value of all comparisons was *r* = 0.642.

**Table 1:** Spearman's rank correlation coefficients (r) for the inter-observer reliability.

**a)**	**Obs 2**	**Obs 3**	**Obs 4**	**Obs 5**	**b)**	**Obs 2**	**Obs 3**	**Obs 4**	**Obs 5**
**Obs 1**	*r* = 0.66, *p* < 0.001	*r* = 0.62, *p* < 0.001	*r* = 0.64, *p* < 0.001	*r* = 0.71, *p* < 0.001	**Obs 1**	*r* = 0.69, *p* < 0.001	*r* = 0.41, *p* = 0.04	*r* = 0.39, *p* = 0.047	*r* = 0.46, *p* = 0.015
**Obs 2**		*r* = 0.70, *p* < 0.001	*r* = 0.67, *p* < 0.001	*r* = 0.65, *p* < 0.001	**Obs 2**		*r* = 0.39, *p* = 0.046	*r* = 0.41, *p* = 0.03	*r* = 0.55, *p* = 0.003
**Obs 3**			*r* = 0.57, *p* < 0.001	*r* = 0.60, *p* < 0.001	**Obs 3**			*r* = 0.67, *p* < 0.001	*r* = 0.49, *p* = 0.01
**Obs 4**				*r* = 0.60, *p* < 0.001	**Obs 4**				*r* = 0.67, *p* < 0.001
	average of all r's = 0.642			average of all r's = 0.514	

Observers (Obs) 1 and 2 tested 50 birds for the feather score, and 27 birds for all other combinations. a) For the plumage assessment, 6 body regions per animal were included (i.e., Obs 1 and 2 tested 50 birds * 6 regions = 300 scores), b) for the fat score.

For the fat score, we obtained slightly different results. The Spearman's rank correlation coefficient showed lower values, which, however, were at least *r* > 0.39 when considering all possible observer pair comparisons (Table 1b). The average r-value of all comparisons was *r* = 0.514. Fat score seems to be a less reliable scoring system than the newly developed plumage feather score and it seems that it requires more experience to obtain a reliable measure.

**Figure 4:**
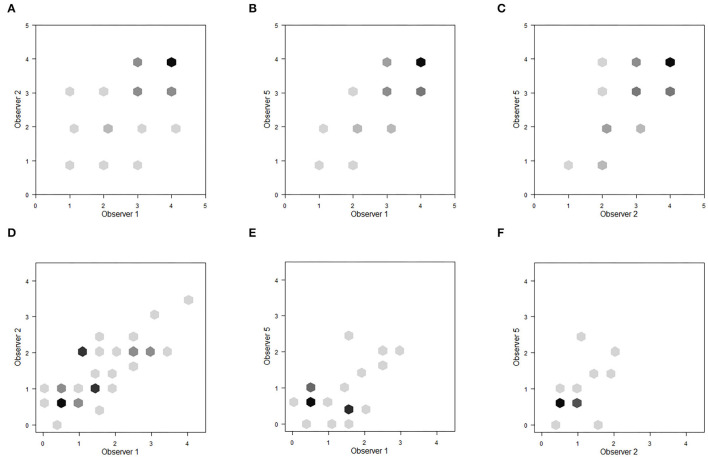
Inter-observer reliability–Score accordance of **(A,D)** observers 1 and 2; **(B,E)** observers 1 and 5; **(C,F)** observers 2 and 5. **(A–C)** Show the plumage scores points for each body part (a: 300 scores, all other 162 scores); **(D–F)** show the fat scores (d: 50 scores, all other 27 scores). Darker points represent overlapping scores, the darker the more overlapping data points.

The inter-observer reliability for the novel plumage score was substantial and it seems to be feasible to quick and easy teach inexperienced observers. In contrast, the fat score from Kaiser (23) seems to be a score for more experienced observers. Figure 4 illustrates the score agreements of selected observer pairs for plumage (Figures 4A–C) and fat (Figures 4D–F); all possible pairs are available in the electronic supplement.

### Intra-Observer Reliability

The intra-observer agreement for the plumage scores was substantially strong (Table 2a). The Spearman's rank correlation for the measurement one vs. five showed a strong and significant agreement (*r* = 0.84, Table 2a). The average r-values of all comparisons of the five measurements were high with *r* = 0.852 (Table 2a).

**Table 2:** Results for intra-observer over the repeated assessments (measurements 1–5, “Meas”) reliability using Spearman's rank correlation coefficients (r).

**a)**	**Meas 2**	**Meas 3**	**Meas 4**	**Meas 5**	**b)**	**Meas 2**	**Meas 3**	**Meas 4**	**Meas 5**
**Meas 1**	*r* = 0.89, *p* < 0.001	*r* = 0.84, *p* < 0.001	*r* = 0.82, *p* < 0.001	*r* = 0.84, *p* < 0.001	**Meas 1**	*r* = 0.94, *p* < 0.001	*r* = 0.91, *p* < 0.001	*r* = 0.90, *p* < 0.001	*r* = 0.90, *p* < 0.001
**Meas 2**		*r* = 0.86, *p* < 0.001	*r* = 0.84, *p* < 0.001	*r* = 0.92, *p* < 0.001	**Meas 2**		*r* = 0.92, *p* < 0.001	*r* = 0.92, *p* < 0.001	*r* = 0.84, *p* < 0.001
**Meas 3**			*r* = 0.84, *p* < 0.001	*r* = 0.84, *p* < 0.001	**Meas 3**			*r* = 0.94, *p* < 0.001	*r* = 0.88, *p* < 0.001
**Meas 4**				*r* = 0.82, *p* < 0.001	**Meas 4**				*r* = 0.85, *p* < 0.001
	average of all r's = 0.852			average of all r's = 0.90	

a) For plumage, where it should be noted that 6 body regions per animal were considered (i.e., 22 birds * 6 regions = 132 scores. b) Fat measurements (22 birds).

For the fat score, a strong agreement (*r* = 0.90; Table 2b) was found when considering measurements one vs. five. The average r-value of all comparisons of the measurements was *r* = 0.90.

**Figure 5:**
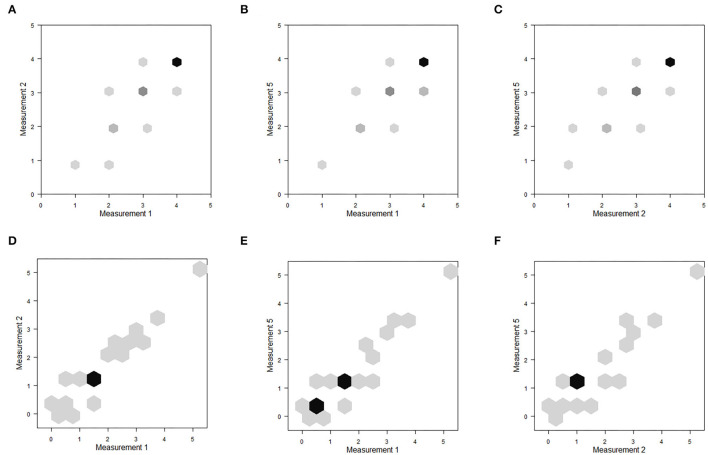
Intra-observer reliability–Score accordance of **(A,D)** measurements 1 and 2; **(B,E)** measurements 1 and 5; **(C,F)** measurements 2 and 5. **(A–C)** show the plumage score points for each body part (132 scores); **(D–F)** show the fat score (22 scores). Darker points represent overlapping scores, the darker the more overlapping data points.

Figure 5 illustrates the score agreements of selected measurement pairs for plumage (Figures 5A–C) and fat (Figures 5D–F); all possible pairs of comparison are shown in the electronic supplement.

## Discussion

Our novel plumage condition score provides a non-invasive tool to easily and reliably assess the plumage condition of small passerines, as shown using the example of the Zebra Finch. It is robust, reliable, and probably easier to teach to others than other body scores, such as the fat score (23). Furthermore, it does cover different condition-dependent parameters of the bird's body.

Assessing non-invasive well-being indicators is quite common in farm animals, including avian species such as domestic chickens, turkeys, or quails (5, 28, 29). Individual-based condition parameters allow to obtain an estimate of the health condition and thus a proxy of the well-being of the animals (6). In a second step, they may also be used in the studies that evaluate different housing conditions as a factor that provides information about how much a given captive condition might be suitable for the animals, while other parameters such as behavior may also be considered. For farmed birds, plumage scores have been commonly used (6, 28), but such score systems were lacking so far for smaller birds such as passerines which are often used as laboratories, zoo, or pet birds. Our plumage condition score can therefore provide a novel tool for small passerines, such as the Zebra Finch, to systematically and reliably assess an individual's plumage status. The plumage score shows a good inter- and intra-observer reliability. Furthermore, in theory, plumage can be assessed without taking the bird out of its housing environment. However, we recommend handling birds briefly and gently. The plumage condition score might represent an interesting tool for future studies that want to assess well-being and/or health conditions in their study designs. Plumage scores may also make sense for a long-term assessment of conditions as molting takes time and also loss or damage of feathers might not appear as fast as other changes in birds. Thus, in contrast to, for example, the fat score, which may change quickly with food availability in small passerines (19), a plumage score is probably more reliable and may better reflect long-term effects. The fat score was less reliable between different observers than the plumage score in our study, but this is not surprising as this score is more complex to assess. Small variation in positioning the bird may cause differences in the scoring (18). Here, an intensive training is necessary to generate reliable measurements. The agreement between the two most experienced observers in fat scores was substantial good, whereas the agreement with an untrained observer was moderate. The intra-observer reliability of a trained observer was quite strong. Plumage scoring, however, can be much more readily taught to new observers. A potential confounding factor that influences fat measures can be the variation in fat storage during the day (19). Since fat reserves vary throughout the day, differences in scoring in succession can also be based on the actual differences and do not have to be an erroneous scoring of the observer. In addition, it should be noted that scoring of different birds should be done at the same time of day, thereby taking diurnal variation into account and thus avoiding an influence of the time of day.

For easy monitoring of health and well-being of birds, it might be suitable to regularly take multiple non-invasive measures of a subset of a population. Besides, the single plumage scores of the different body regions also the overall plumage damage score could represent a key parameter, which could or should be accompanied by others such as body weight and may be also fat score. Specifically, plumage scores and weight might be robust against observer effects, as plumage scoring seems to be quite easy to learn and body weight can be obtained accurately using a balance, whereas only plumage scores are robust against daily variations appearing in body mass and body fat. For routine animal monitoring in stock populations, an alarm signal may be if birds of one housing group have systematically lower plumage scores than others. This would require the animal owner or caretaker to have a more detailed look with respect to the proposed plumage score of these birds. The plumage scores provide direct information about the bird's feather status, and a poor plumage may have direct effects for the bird. A plucked plumage can entail disadvantages in temperature regulation (30) and may reduce flight abilities and lead to reduced attractiveness.

In conclusion, we propose the plumage scoring system, with the resulting cumulative plumage damage score, for small passerines, such as the Zebra Finch, as a reliable new tool to assess well-being and health in birds non-invasively. Future studies should examine the direct link of the plumage scores to well-being, welfare, and health.

## Data Availability Statement

The raw data supporting the conclusions of this article will be made available by the authors, without undue reservation upon request to the corresponding author.

## Author Contributions

OK: providing resources. LK: statistical analysis and investigation, data curation, writing original draft, and creating figures. LK, OK, and ETK: conceptualization and methodology, writing, review and editing manuscript, and idea. All authors contributed to the article and approved the submitted version.

## Conflict of Interest

The authors declare that the research was conducted in the absence of any commercial or financial relationships that could be construed as a potential conflict of interest.

## Publisher's Note

All claims expressed in this article are solely those of the authors and do not necessarily represent those of their affiliated organizations, or those of the publisher, the editors and the reviewers. Any product that may be evaluated in this article, or claim that may be made by its manufacturer, is not guaranteed or endorsed by the publisher.

## References

[1] Dudde A Weigend S Krause ET Jansen S Habig C Schrader L. Chickens in motion: Effects of egg production level and pen size on the motor abilities and bone stability of laying hens (Gallus gallus forma domestica). Appl Anim Behav Sci. (2020) 227:104998. 10.1016/j.applanim.2020.104998

[2] Kestin SC Gordon S Su G Soerenson P. Relationships in broiler chickens between lameness, liveweight, growth rate and age. Vet Rec. (2001) 148:195–8. 10.1136/vr.148.7.195 11265995

[3] Kestin SC Knowles TG Tinch AE Gregory NG. Prevalence of leg weakness in broiler chickens and its relationship with genotype. Vet Rec. (1992) 131:190–4. 10.1136/vr.131.9.190 1441174

[4] Malchow J Dudde A Berk J Krause ET Sanders O Puppe B . Is the rotarod test an objective alternative to the gait score for evaluating walking ability in chickens? Animal Welfare. (2019) 28:261–9. 10.7120/109627286.28.3.261

[5] Bilčík B Keeling LJ. Changes in feather condition in relation to feather pecking and aggressive behaviour in laying hens. Br Poult Sci. (1999) 40:444–51. 10.1080/00071669987188 10579400

[6] Blokhuis HJ Fiks Van Niekerk T Bessei W Elson A Guémené D Kjaer JB . The LayWel project: Welfare implications of changes in production systems for laying hens. Worlds Poult Sci J. (2007) 63:101–14. 10.1017/S0043933907001328

[7] Griffith SC Buchanan KL. The Zebra Finch: the ultimate Australian supermodel. Emu. (2010) 110:v–vii. 10.1071/MUv110n3_ED

[8] Castellini C Perella F Mugnai C dal Bosco A. Welfare, productivity and qualitative traits of egg in laying hens reared under different rearing systems. In: XII European Polutry Conference (2006).

[9] Campo JL Gil MG Torres O Davila SG. Association between plumage condition and fear and stress levels in five breeds of chickens. Poult Sci J. (2001) 80:549–52. 10.1093/ps/80.5.549 11372702

[10] Nätt D Kerje S Andersson L Jensen P. Plumage color and feather pecking—behavioral differences associated with PMEL17 genotypes in chicken (*Gallus gallus*). Behav Genet. (2007) 37:399–407. 10.1007/s10519-006-9125-0 17106652

[11] Krause ET Naguib M. Zebra finch males compensate in plumage ornaments at sexual maturation for a bad start in life. Front Zool. (2015) 12:S11. 10.1186/1742-9994-12-S1-S11 26816511 PMC4722338

[12] Naguib M Nemitz A. Living with the past: nutritional stress in juvenile males has immediate effects on their plumage ornaments and on adult attractiveness in zebra finches. PLoS ONE. (2007) 2:e901. 10.1371/journal.pone.0000901 17878936 PMC1975674

[13] Peters A Delhey K Johnsen A Kempenaers B. The condition-dependent development of carotenoid-based and structural plumage in nestling blue tits: Males and females differ. American Naturalist. (2007) 169:122–35. 10.1086/510139 29517928

[14] Krause ET Ruploh T. Captive domesticated zebra finches (Taeniopygia guttata) have increased plasma corticosterone concentrations in the absence of bathing water. Appl Anim Behav Sci. (2016) 182:80–5. 10.1016/j.applanim.2016.06.003

[15] Gosler AG. Environmental and social determinants of winter fat storage in the great tit parus major. J Anim Ecol. (1996) 65:1–17. 10.2307/5695

[16] Maggini I Bairlein F. Body condition and stopover of trans-Saharan spring migrant passerines caught at a site in southern Morocco. Ring Migr. (2011) 26:31–7. 10.1080/03078698.2011.586591

[17] Meijer T Möhring FJ Trillmich F. Annual and daily variation in body mass and fat of starlings sturnus vulgaris. J Avian Biol. (1994) 25:98–104. 10.2307/3677026

[18] Rogers CM. An evaluation of the method of estimating body fat in birds by quantifying visible subcutaneous fat. J Field Ornithol. (1991) 62:349–56.

[19] Rozman J Meijer T. Daily variation of body mass and fat reserves in Australian Zebra Finches (Taeniopygia guttata castanotis). Biological Conservation of Fauna. (1998) 102:320–6.

[20] Dickens MJ Delehanty DJ Romero LM. Stress and translocation: alterations in the stress physiology of translocated birds. Proc R Soc B Biol Sci. (2009) 276:2051–6. 10.1098/rspb.2008.1778 19324794 PMC2677253

[21] Ferket PR Gernat AG. Factors That affect feed intake of meat birds: a review. Int J Poult Sci. (2006) 5:905–11. 10.3923/ijps.2006.905.911

[22] German AJ. Weight management in obese pets: the tailoring concept and how it can improve results. Acta Vet Scand. (2016) 58:57. 10.1186/s13028-016-0238-z 27766974 PMC5073926

[23] Kaiser A. A new multi-category classification of subcutaneous fat deposits of songbirds. J Field Ornithol. (1993) 64:246–55.

[24] Forstmeier W Segelbacher G Mueller JC Kempenaers B. Genetic variation and differentiation in captive and wild zebra finches (Taeniopygia guttata). Mol Ecol. (2007) 16:4039–50. 10.1111/j.1365-294X.2007.03444.x 17894758

[25] Hoffman J Krause ET Lehmann K Krüger O. MC1R genotype and plumage colouration in the zebra finch (Taeniopygia guttata): Population structure generates artefactual associations. PLoS ONE. (2014) 9:e86519. 24489736 10.1371/journal.pone.0086519 PMC3906038

[26] R Core Team (2021). R: A Language and Environment for Statistical ComputingNo Title. Available online at: https://www.r-project.org/.

[27] Carr D, ported by Lewin-Koh N, Maechler M, and contains copies of lattice functions written by Deepayan Sarkar. hexbin: Hexagonal Binning Routines. R package version 1.28.2 (2021). Available online at: https://CRAN.R-project.org/package=hexbin.

[28] Dalton HA Wood BJ Widowski TM Guerin MT Torrey S. Changes in leg health, skin, and plumage condition in domestic male turkeys of varying body weights. Appl Anim Behav Sci. (2016) 178:40–50. 10.1016/j.applanim.2016.02.010

[29] Mohammed HH Said EN Abdel-Hamid SE. Impact of different litter materials on behaviour, growth performance, feet health and plumage score of Japanese quail (Coturnix japonica). European Poultry Science. (2017) 81:719–27. 10.1399/eps.2017.172

[30] Richards SA. The influence of loss of plumage on temperature regulation in laying hens. J Agric Sci. (1977) 89:393–8. 10.1017/S0021859600028318

