# Supplementary material for: Plumage and Fat Condition Scores as Well-Being Assessment Indicators in a Small Passerine Bird, the Zebra Finch (Taeniopygia guttata)
Source: Front Vet Sci. 2022 Apr 14;9:791412. doi: 10.3389/fvets.2022.791412 (PMC9048790; doi:10.3389/fvets.2022.791412)
Supplement: Supplementary file 1 [file Table_1.docx]

Electronic Supplement

**Plumage and fat condition scores as well-being assessment indicators in a small passerine bird, the Zebra Finch (*Taeniopygia guttata*)**

**Lisa Kalnins^1,*^, Oliver Krüger^1^, E. Tobias Krause^1,2^**

^1^ Department of Animal Behaviour, Bielefeld University, Konsequenz 45, 33615 Bielefeld, Germany

^2^ Institute of Animal Welfare and Animal Husbandry, Friedrich-Loeffler-Institut, Dörnbergstr. 25/27, 29223 Celle, Germany

* Corresponding author: [lisa.kalnins@uni-bielefeld.de](mailto:lisa.kalnins@uni-bielefeld.de)

| 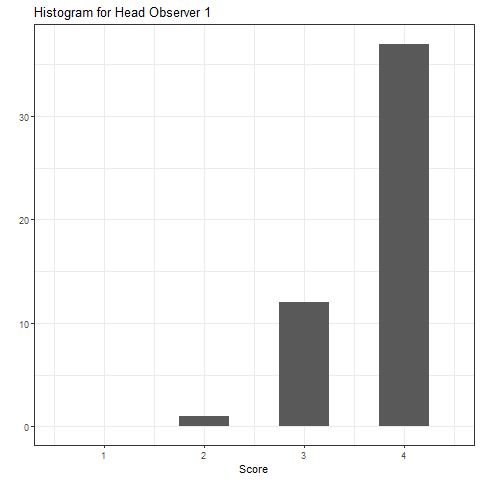 | 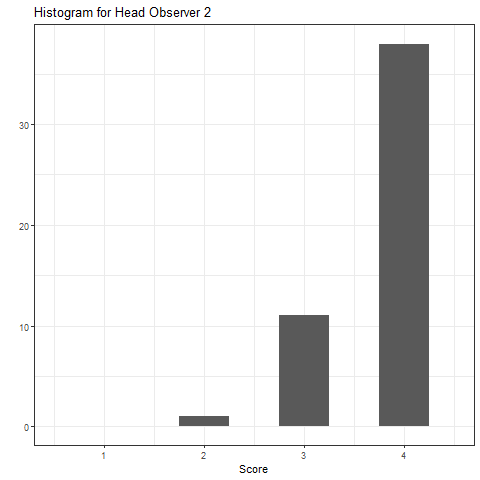 |
| --- | --- |
| 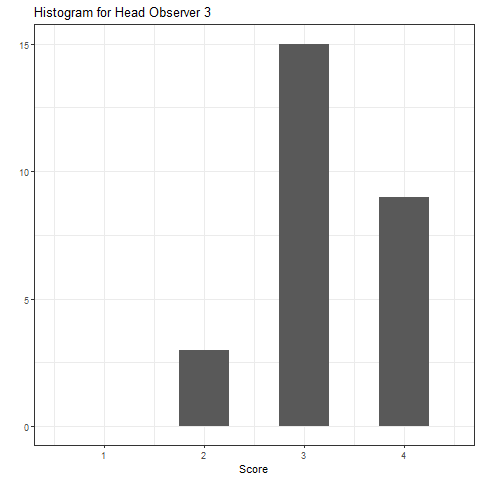 | 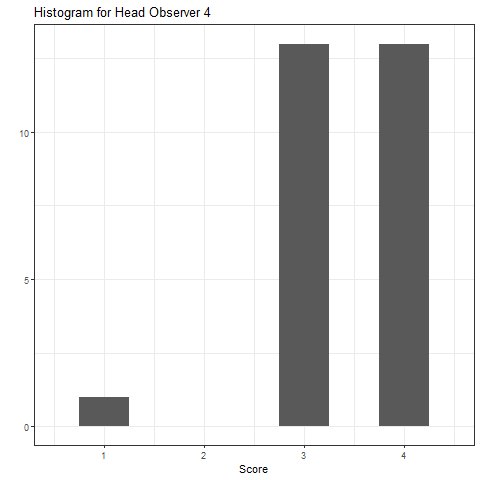 |
| 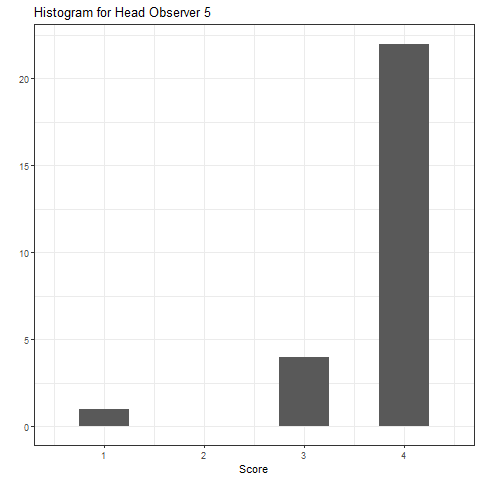 |  |

S1- A: Interobserver reliaility: Histogramm of single scores “head” of each observer

| 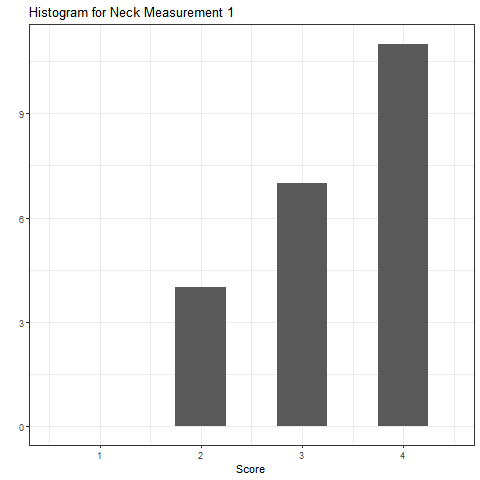 | 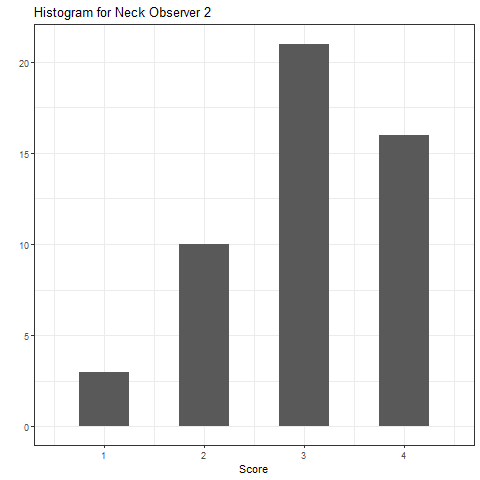 |
| --- | --- |
| 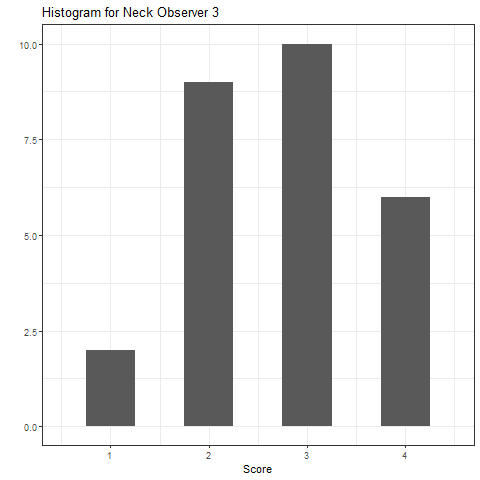 | 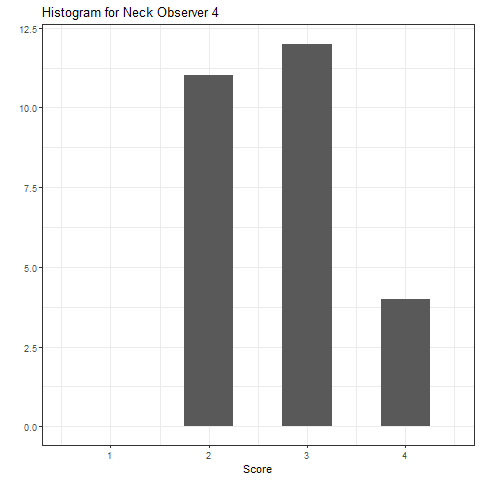 |
| 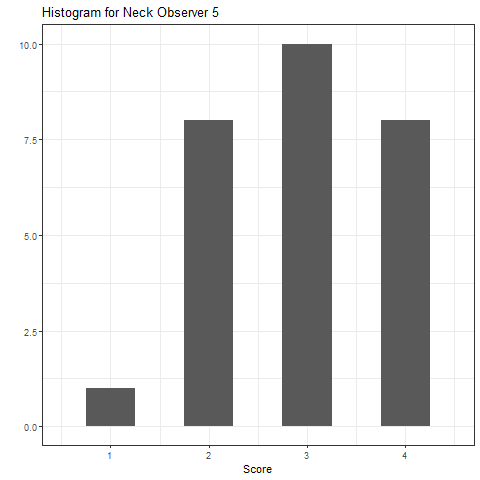 |  |

S1- B: Interobserver reliaility: Histogramm of single scores “neck” of each observer

| 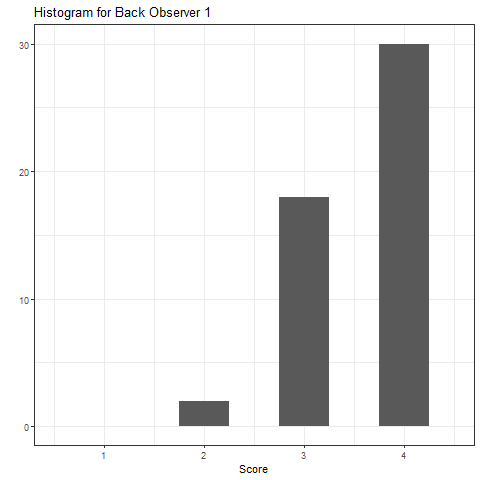 | 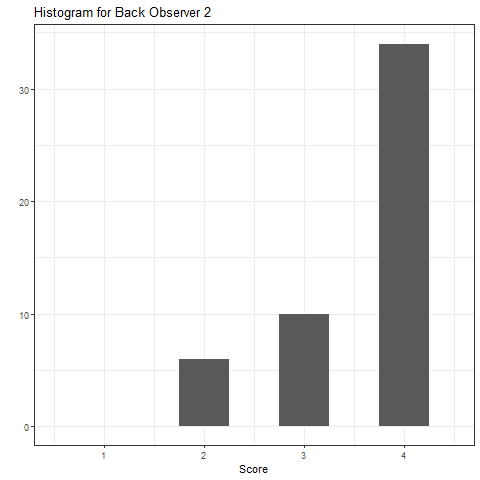 |
| --- | --- |
| 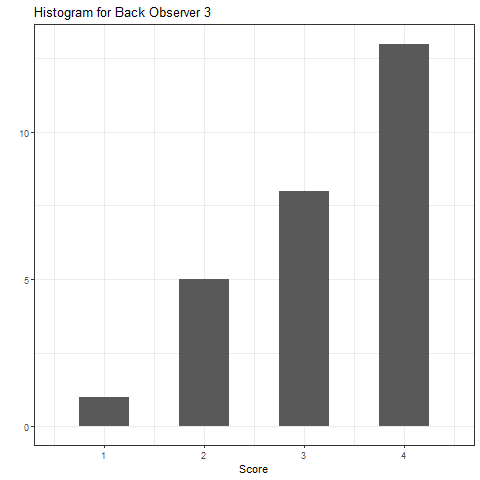 | 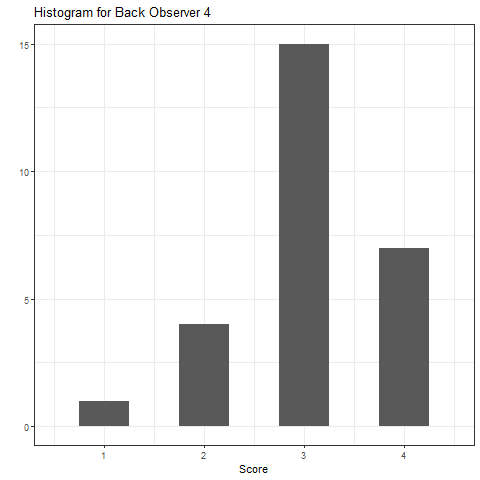 |
| 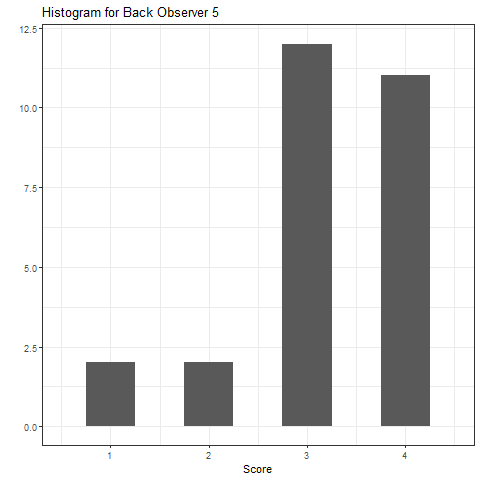 |  |

S1- C: Interobserver reliaility: Histogramm of single scores “back” of each observer

| 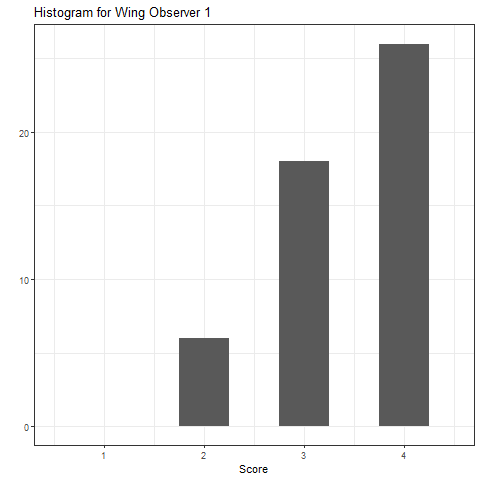 | 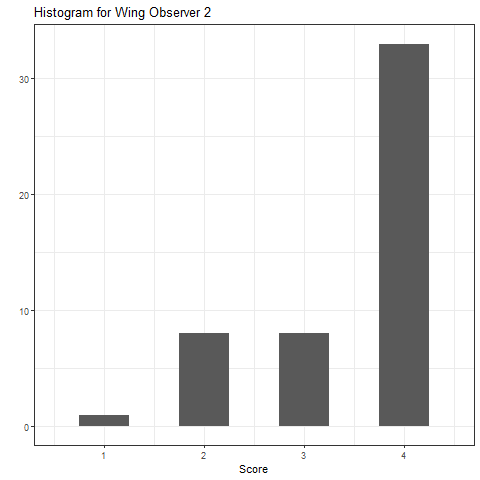 |
| --- | --- |
| 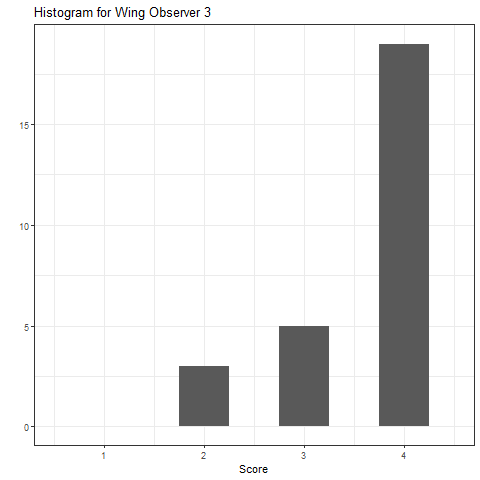 | 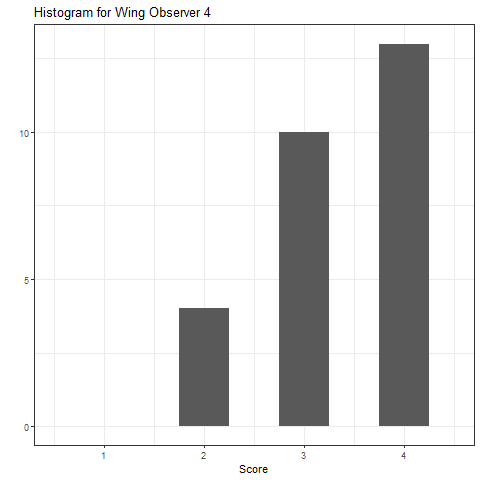 |
| 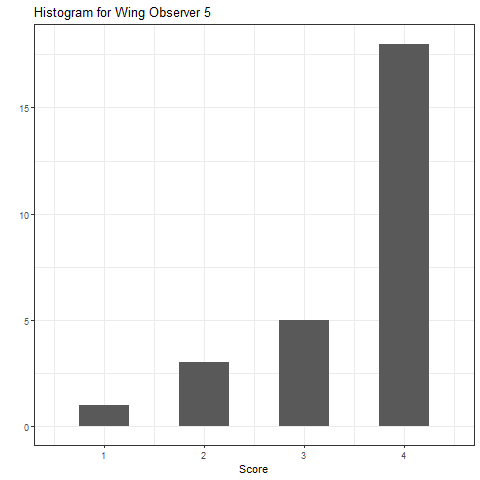 |  |

S1-D: Interobserver reliaility: Histogramm of single scores “wing” of each observer

| 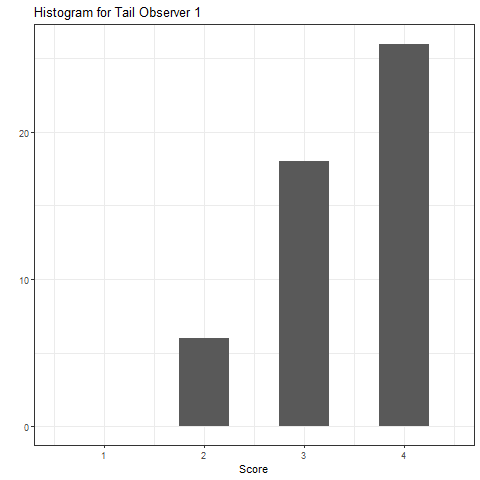 | 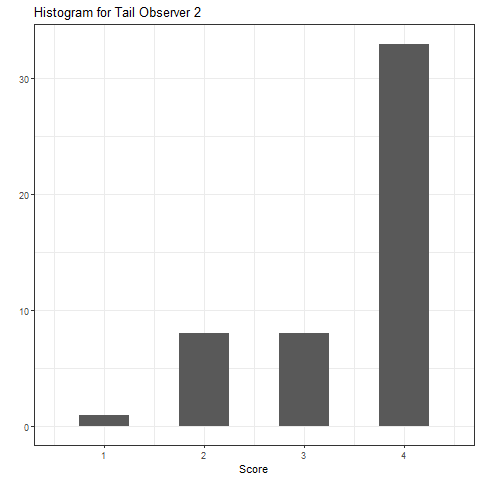 |
| --- | --- |
| 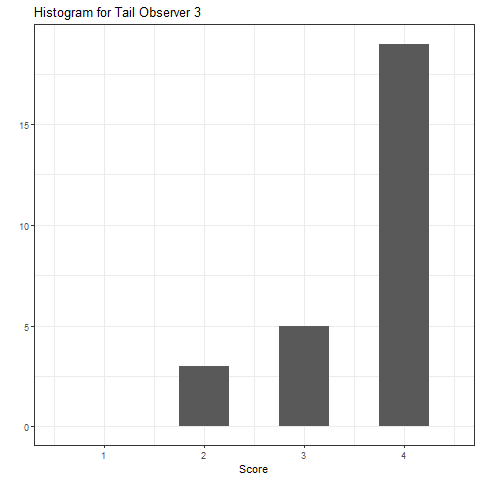 | 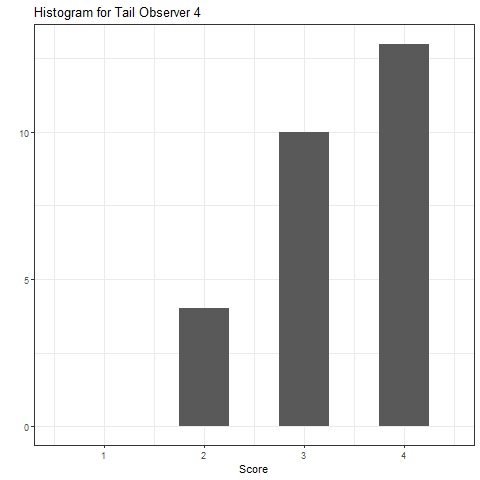 |
| 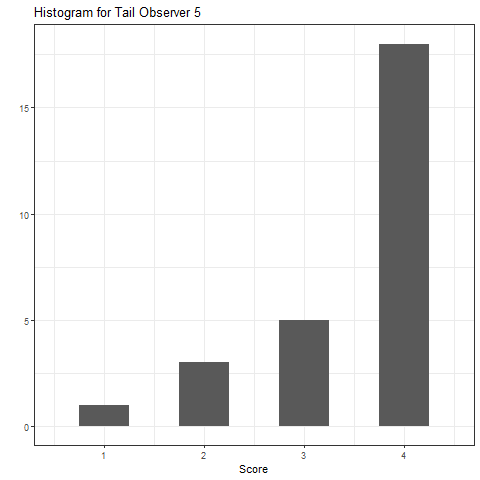 |  |

S1- E: Interobserver reliaility: Histogramm of single scores “tail” of each observer

| 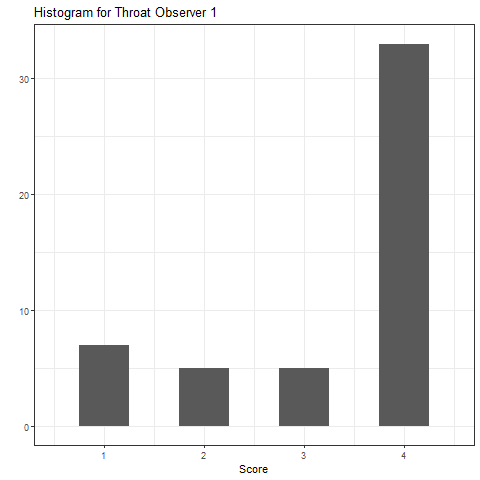 | 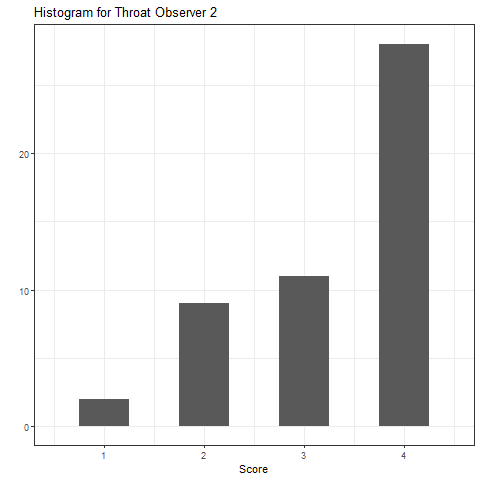 |
| --- | --- |
| 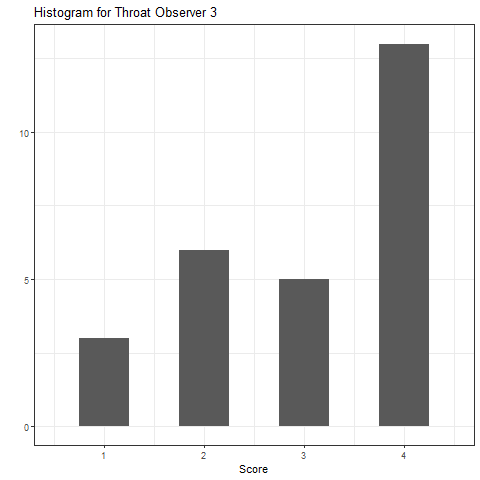 | 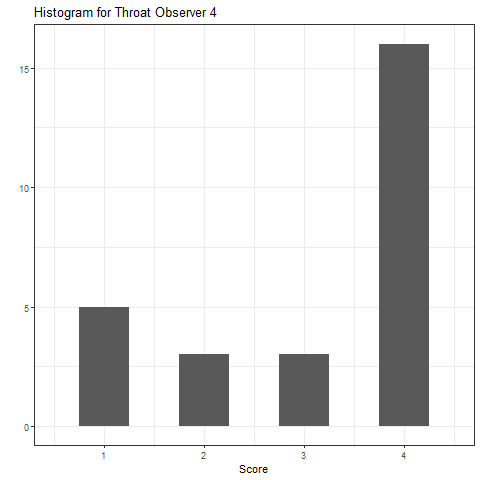 |
| 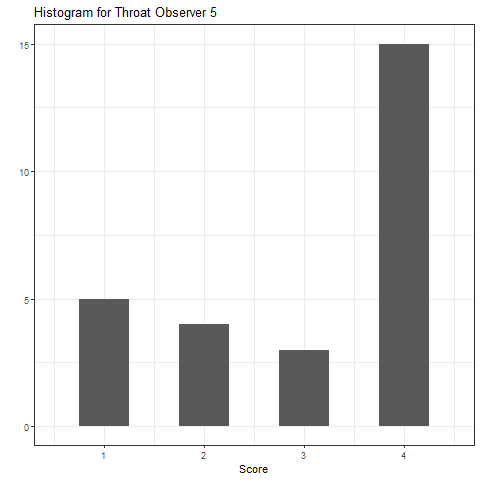 |  |

S1- F: Interobserver reliaility: Histogramm of single scores “thorat” of each observer

| 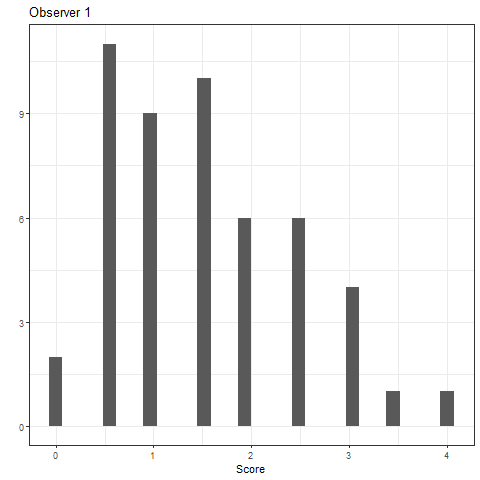 | 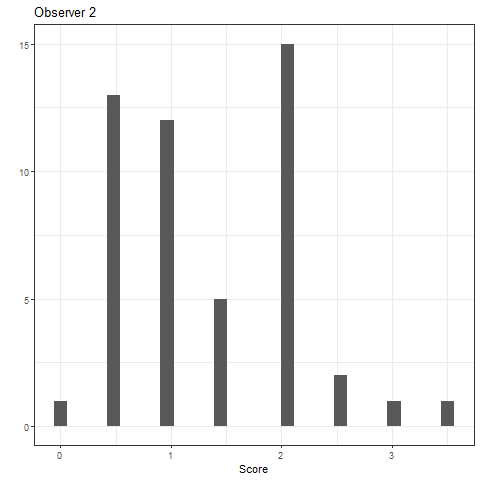 |
| --- | --- |
| 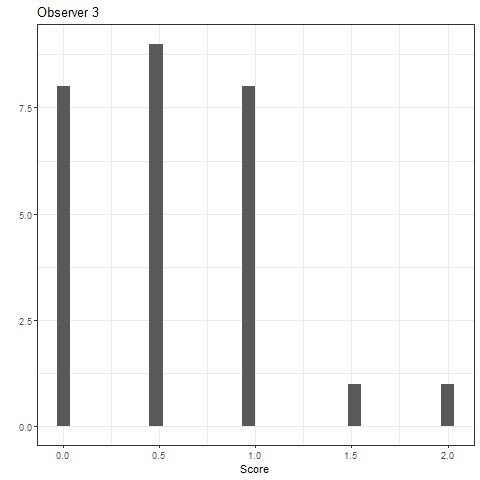 | 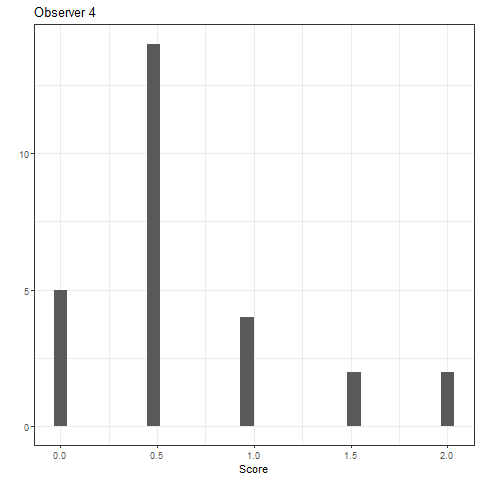 |
| 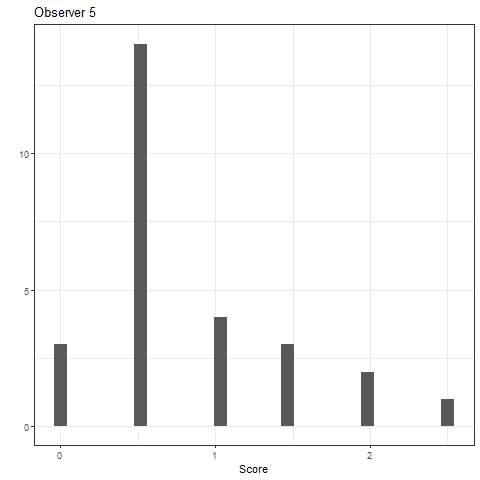 |  |

Supplement 2– Histogramms of the fat score at each body part at each measurement.

| 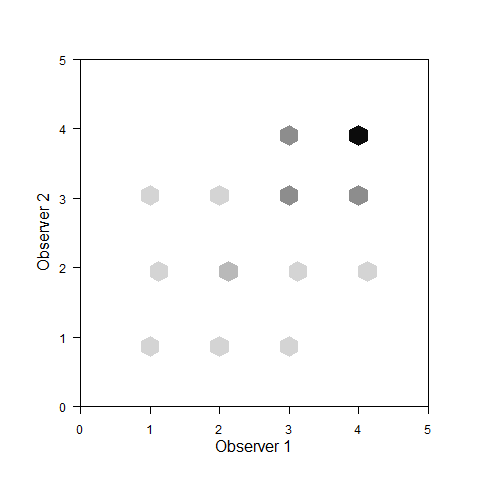 | 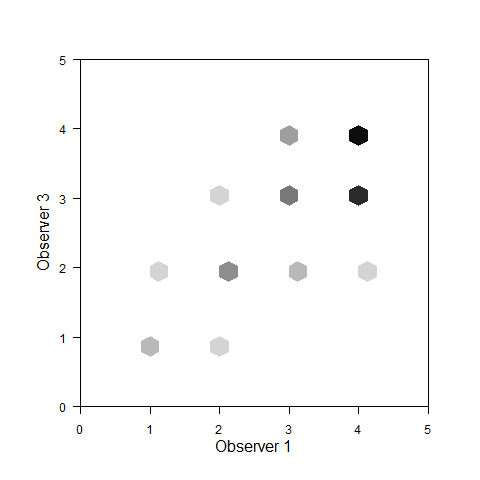 | 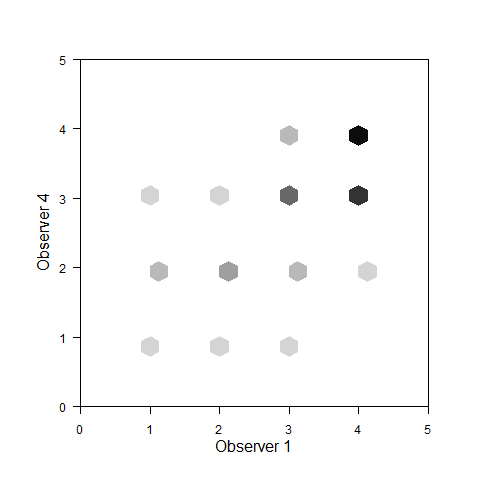 |
| --- | --- | --- |
| 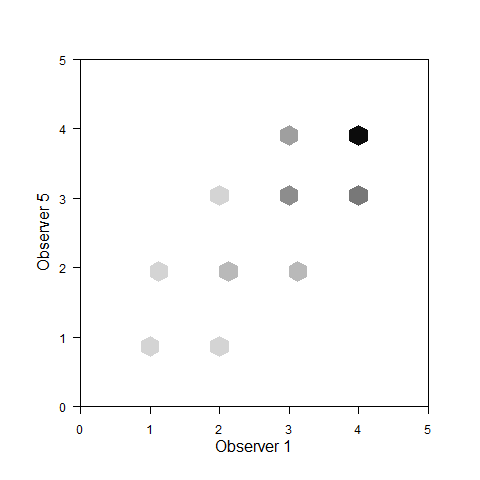 | 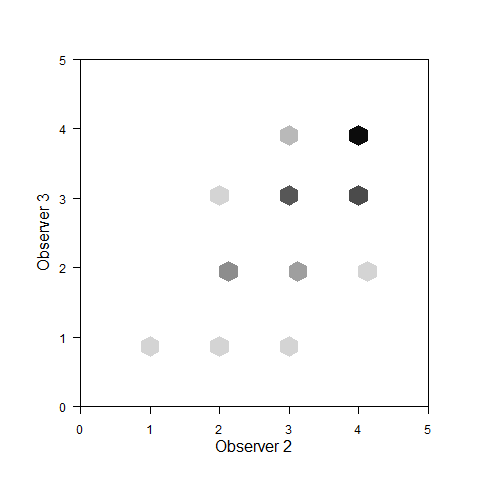 | 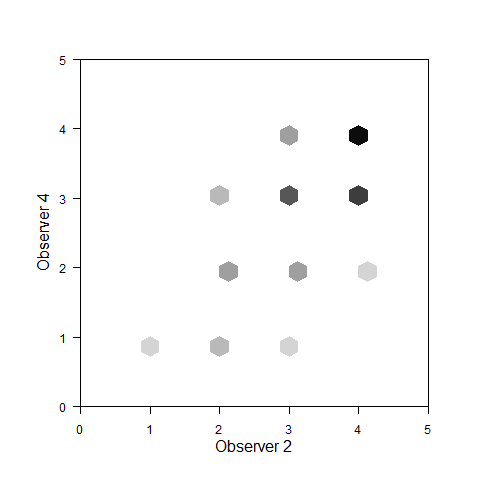 |
| 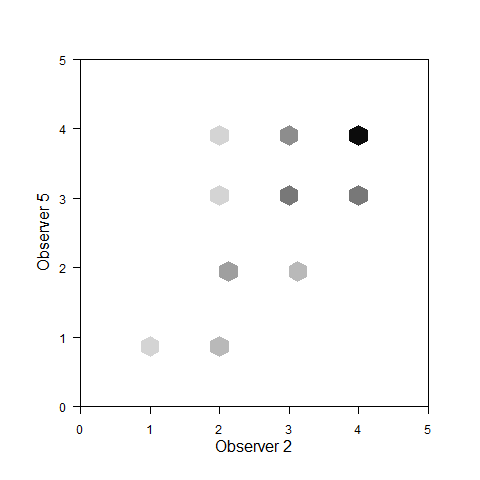 | 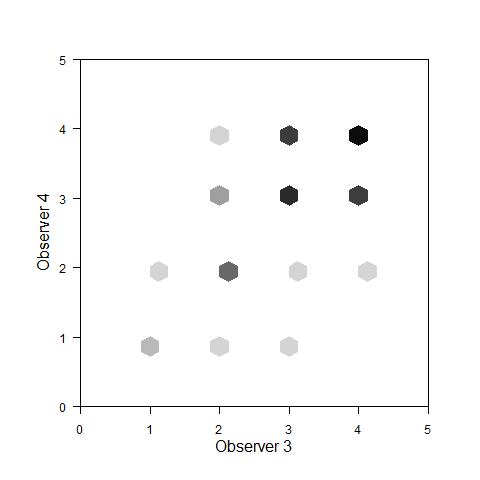 | 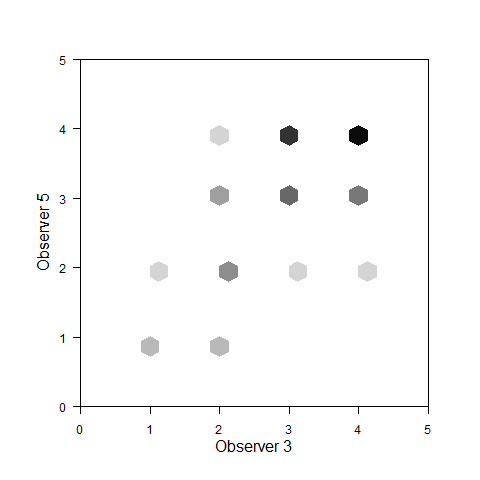 |
| 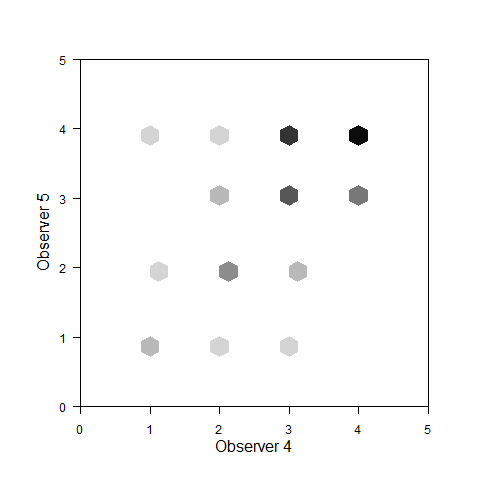 |  |  |

Supplement 3: Body part plumage - score accordance and discordance of all possible observer pairs. Darker points represent more overlapping scorings.

| 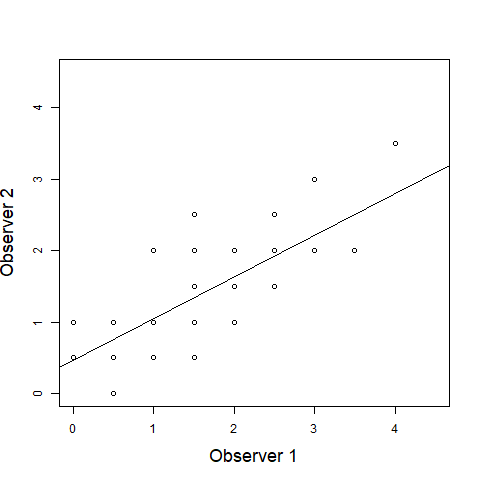 | 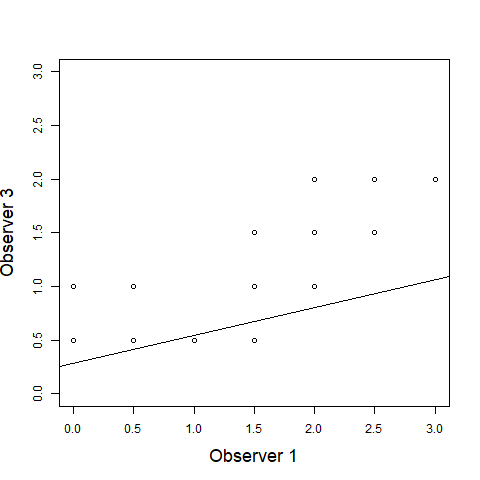 | 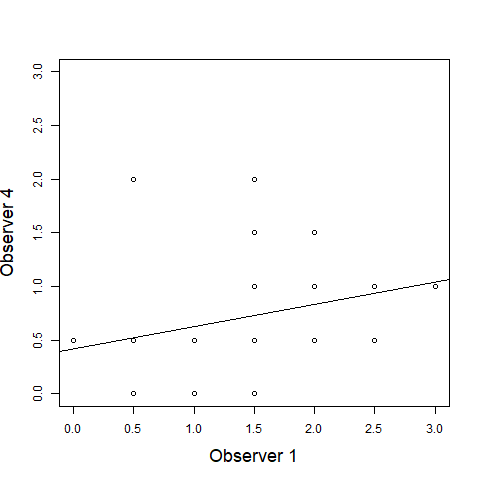 |
| --- | --- | --- |
| 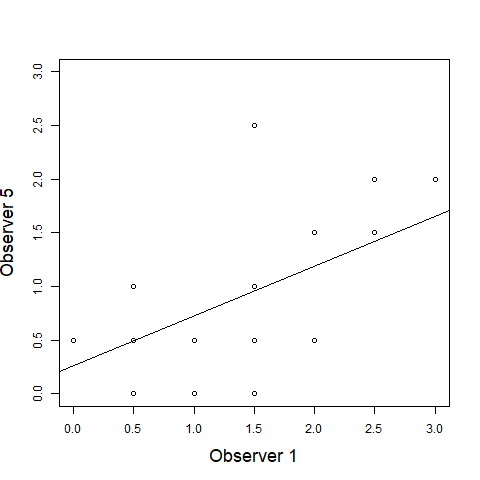 | 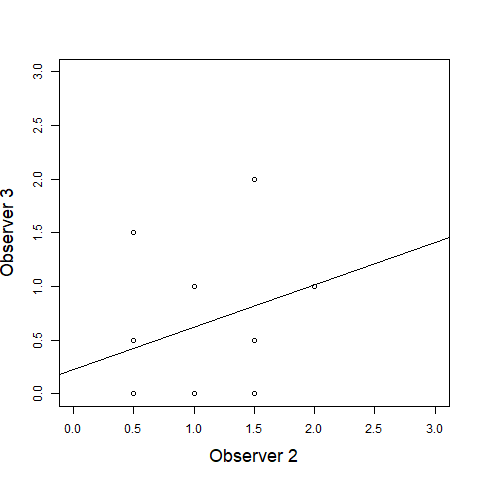 | 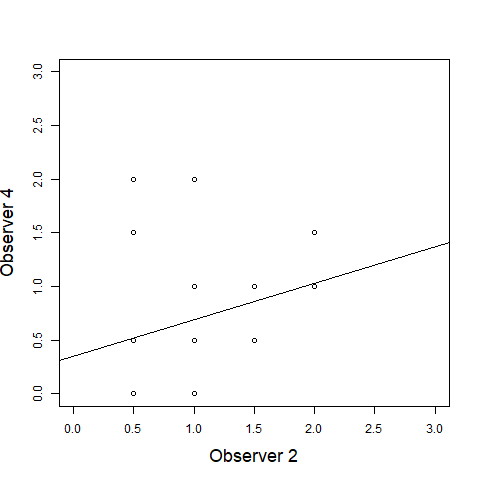 |
| 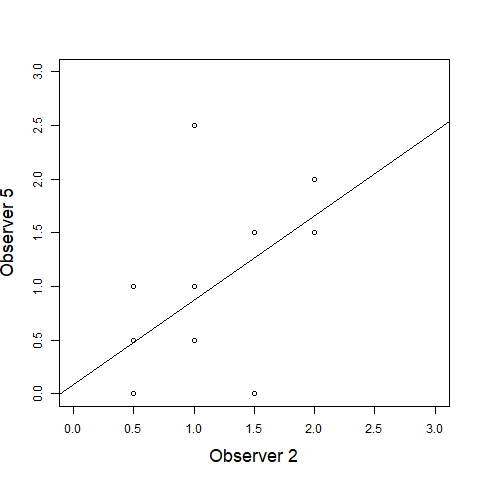 | 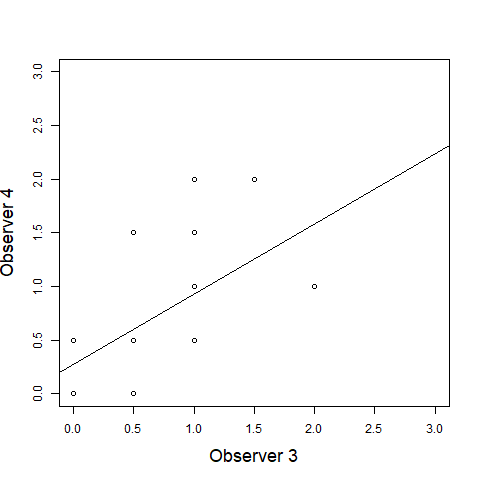 | 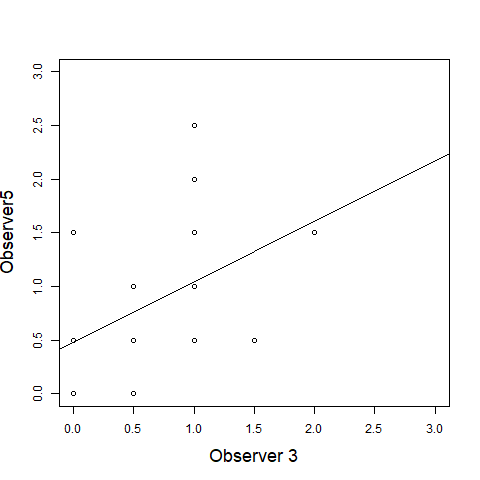 |
| 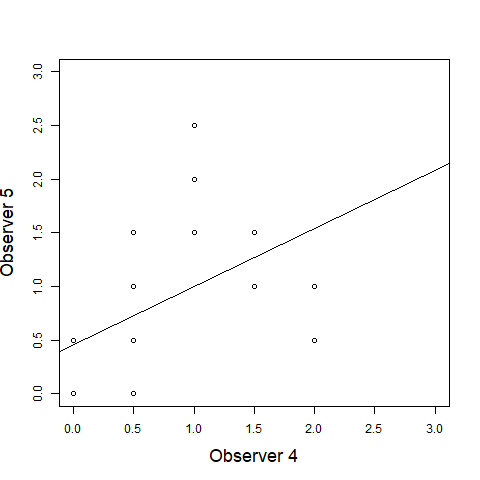 |  |  |

Supplement 4– Fat- score accordance and discordance of all possible observer pairs.

| 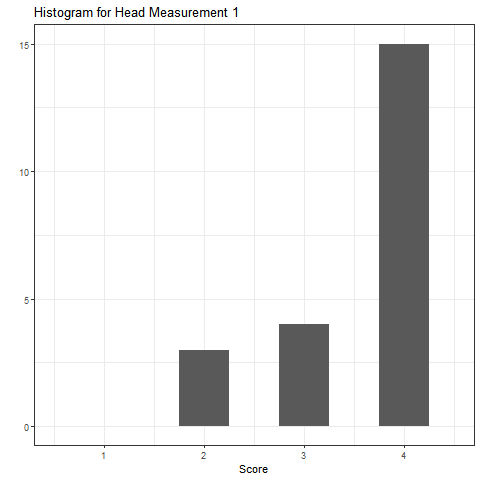 | 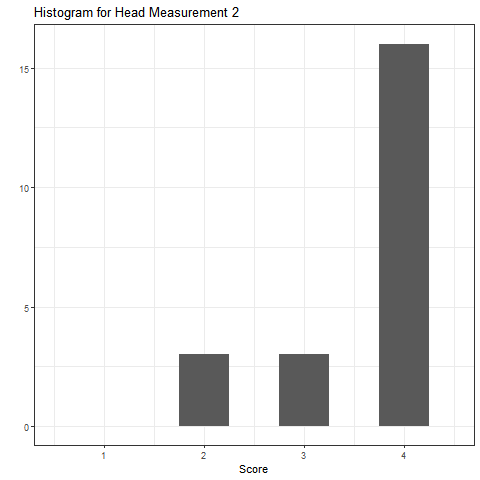 |
| --- | --- |
| 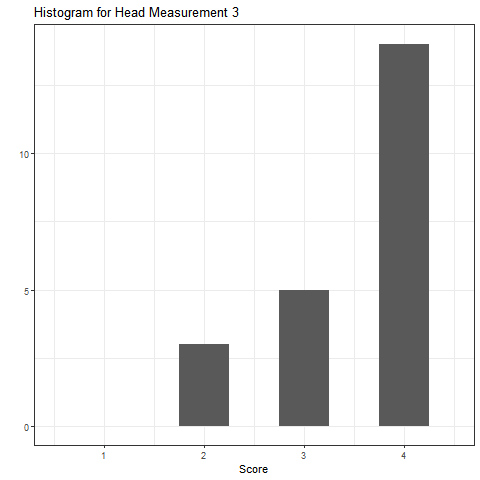 | 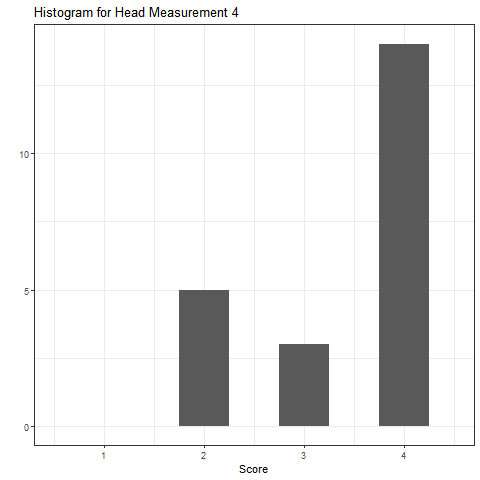 |
| 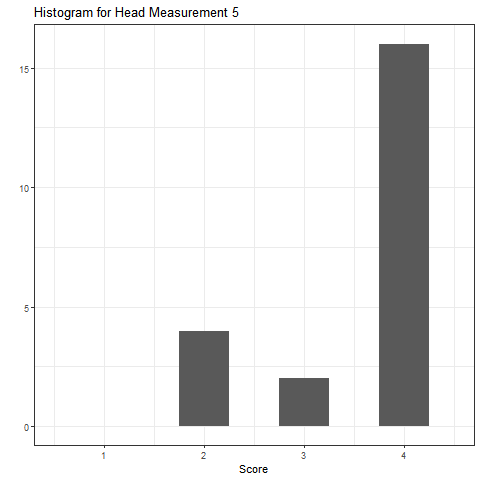 |  |

S5- A: Intra-observer reliaility: Histogramm of single scores “head” of each measurement

| 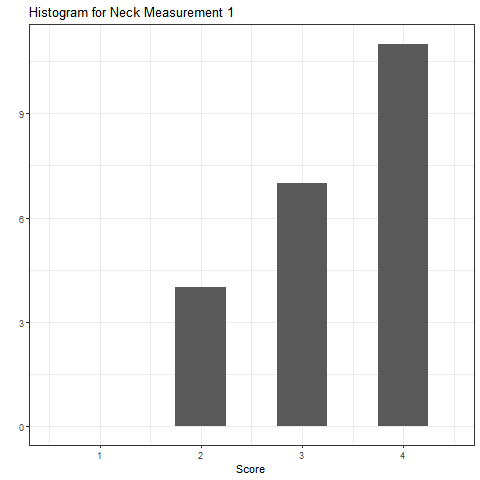 | 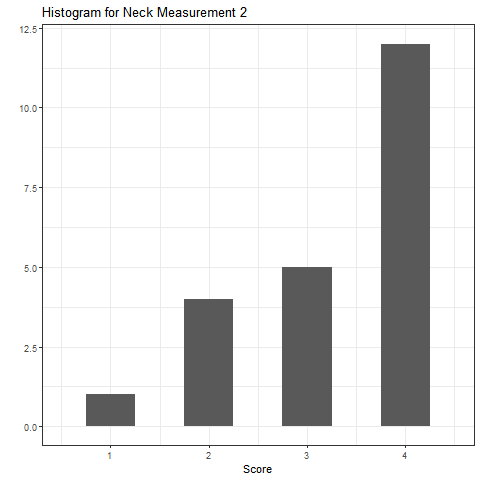 |
| --- | --- |
| 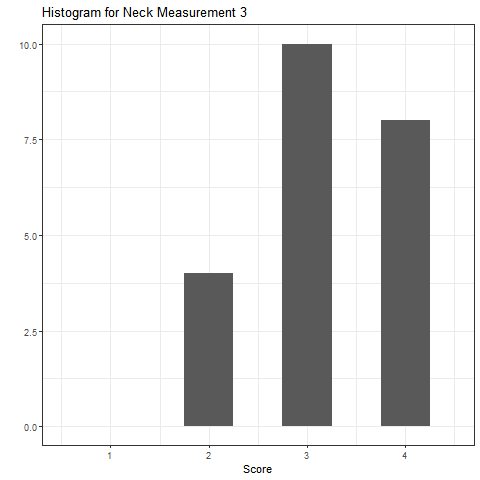 | 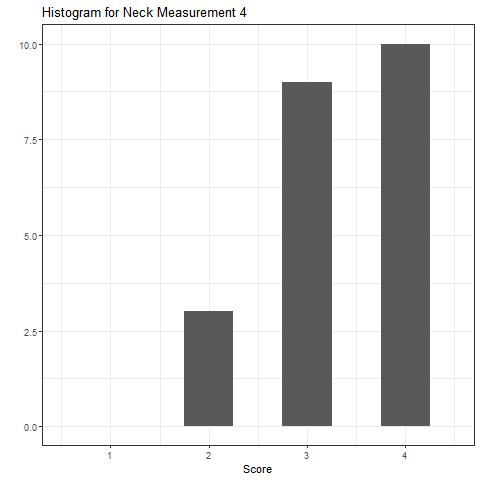 |
| 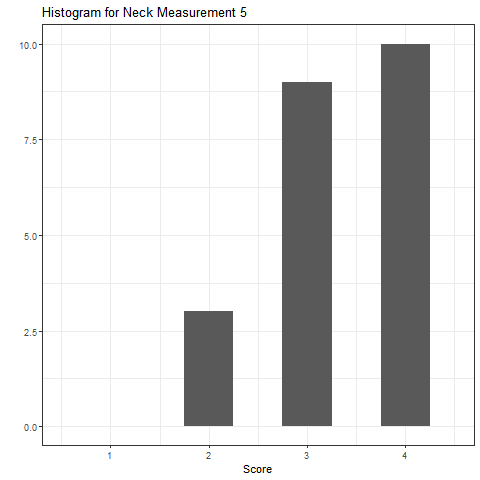 |  |

S5- B: Intra-observer reliaility: Histogramm of single scores “neck” of each measurement

| 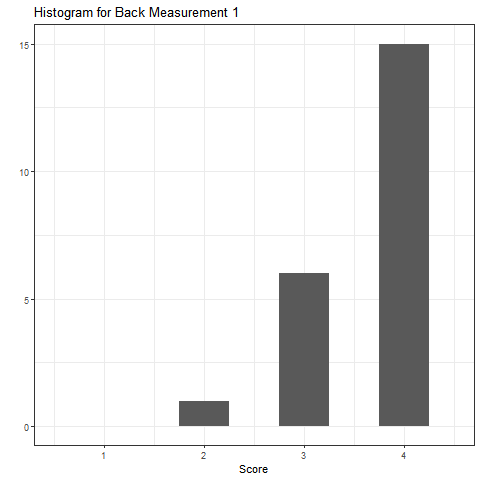 | 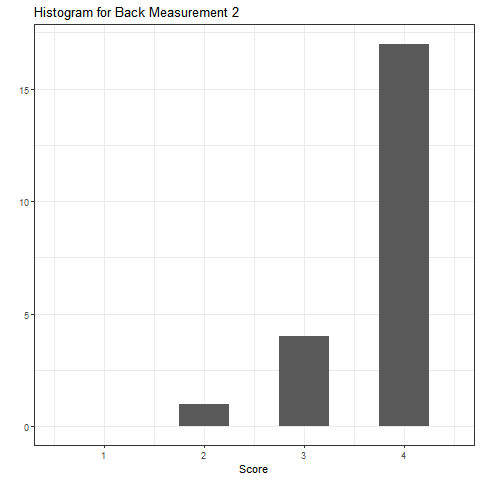 |
| --- | --- |
| 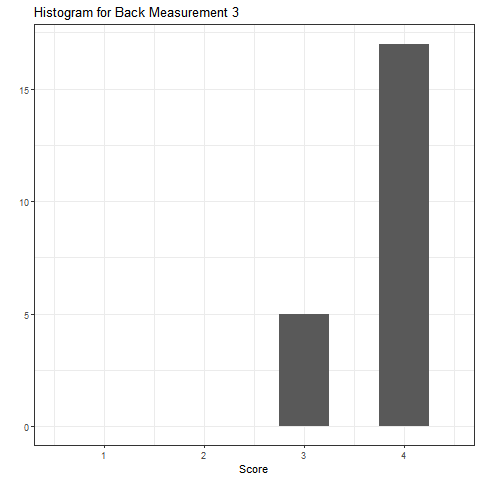 | 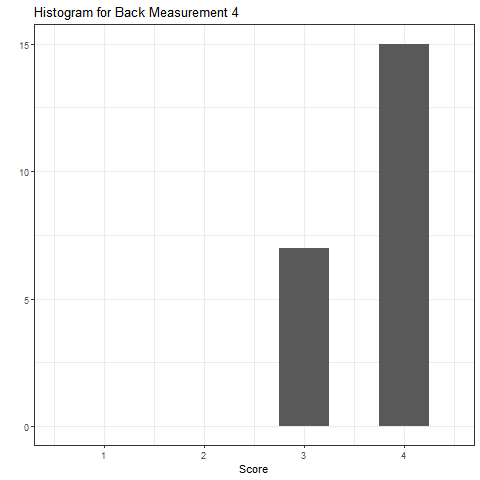 |
| 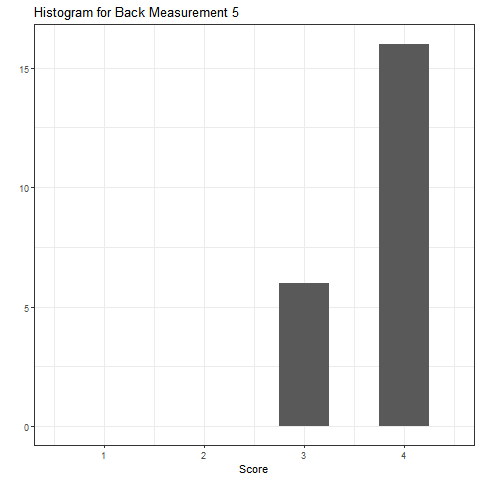 |  |

S5- C: Intra-observer reliaility: Histogramm of single scores “head” of each measurement

| 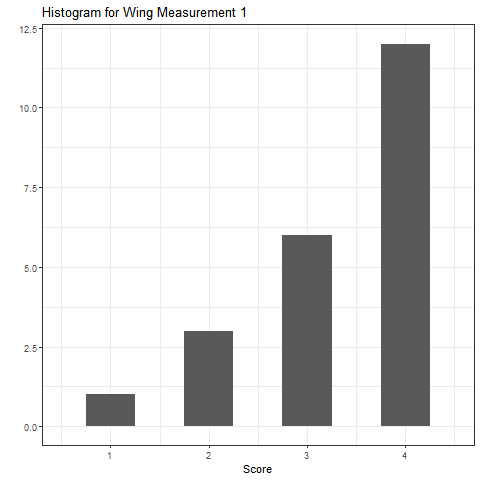 | 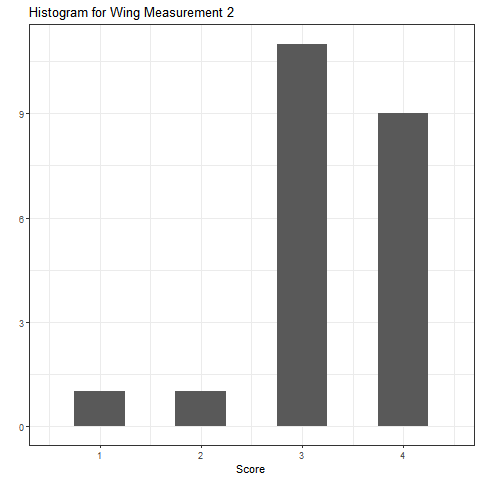 |
| --- | --- |
| 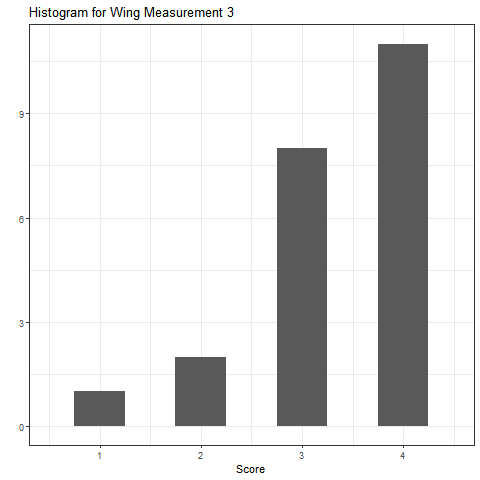 | 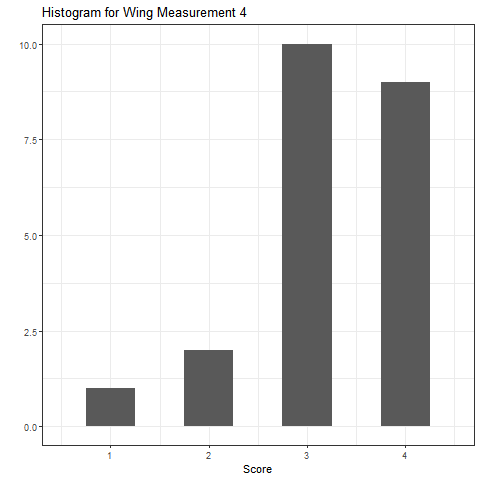 |
| 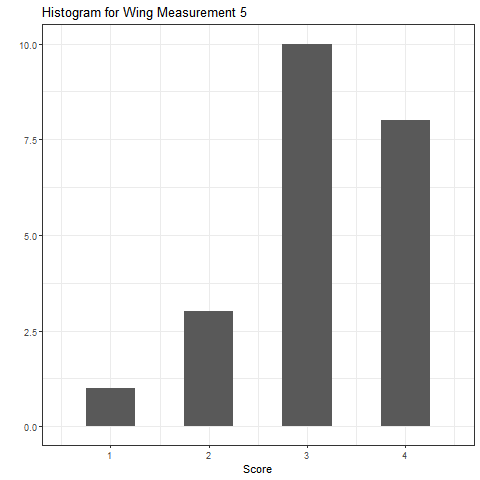 |  |

S5- D: Intra-observer reliaility: Histogramm of single scores “wing” of each measurement

| 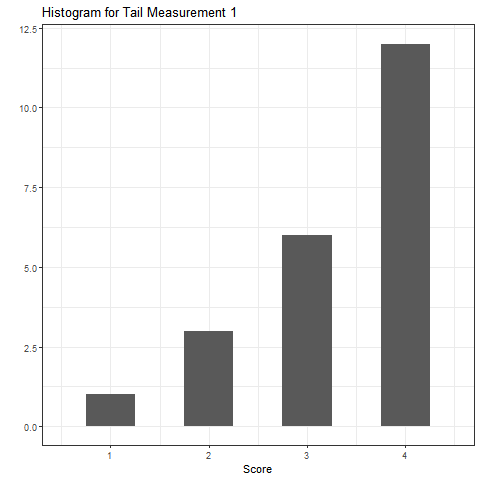 | 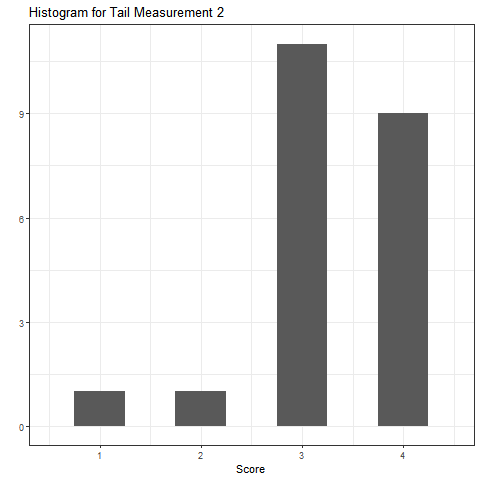 |
| --- | --- |
| 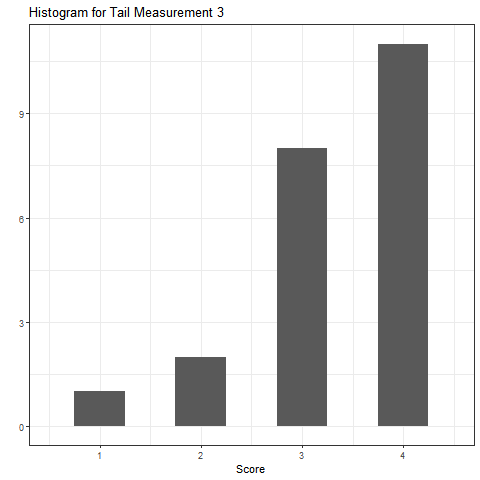 | 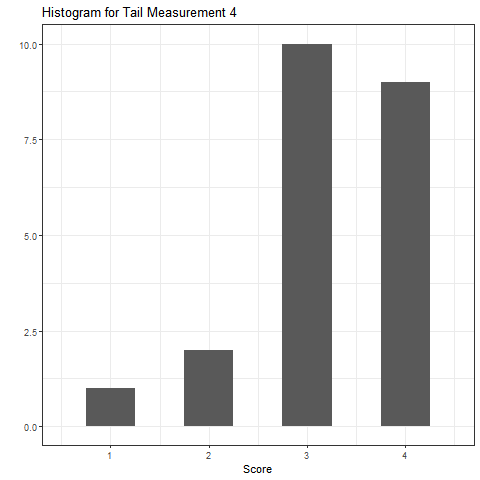 |
| 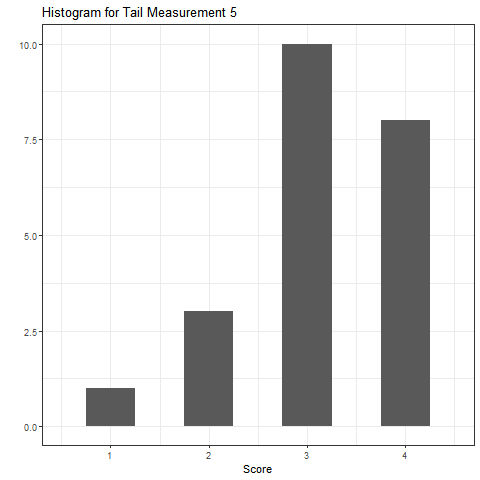 |  |

S5- E: Intra-observer reliaility: Histogramm of single scores “tail” of each measurement

| 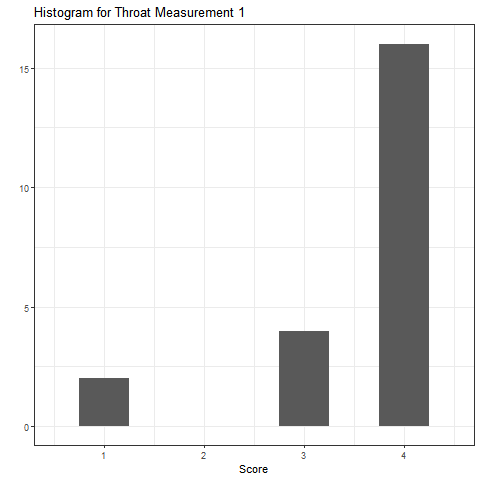 | 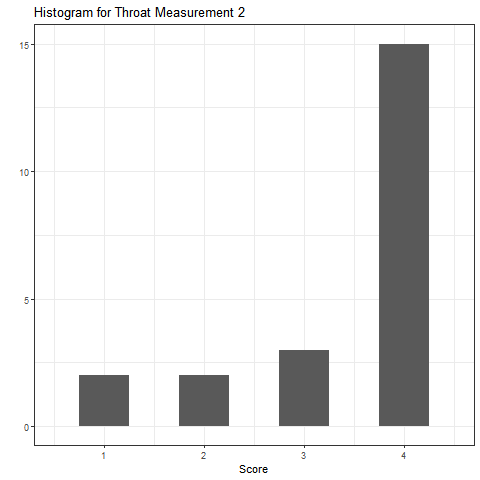 |
| --- | --- |
| 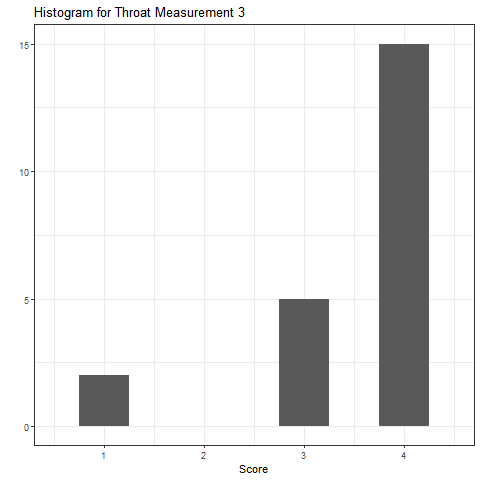 | 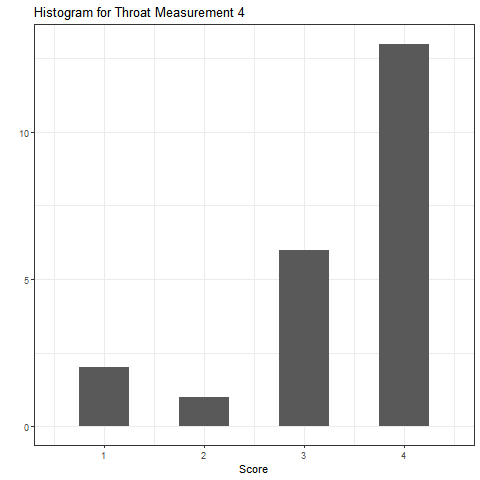 |
| 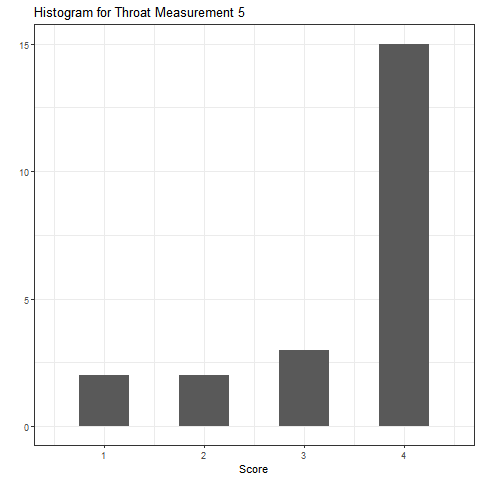 |  |

S5- F: Intra-observer reliaility: Histogramm of single scores “throat” of each measurement

| 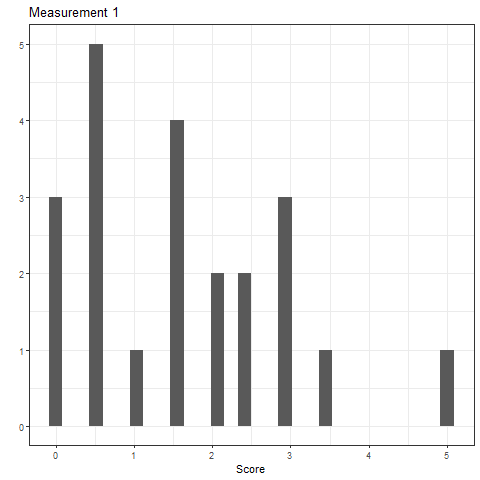 | 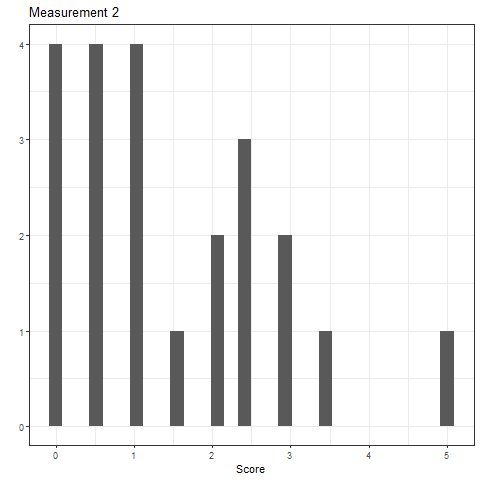 |
| --- | --- |
| 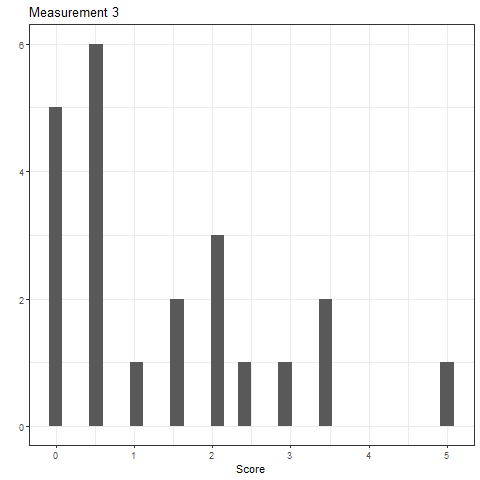 | 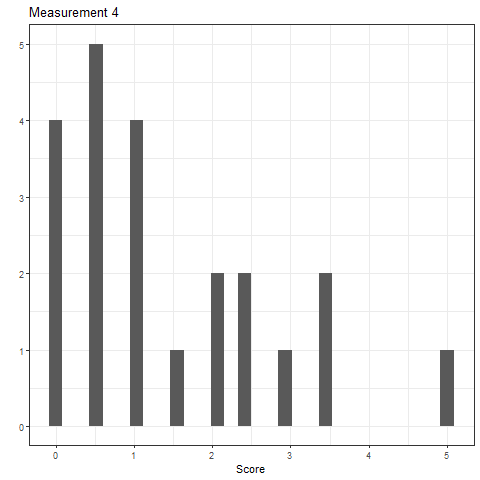 |
| 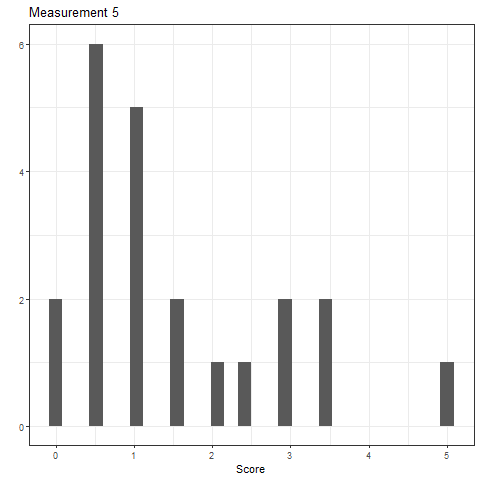 |  |

Supplement 6: Intra-observer reliability – Histogramms of the fat score at each measurement.

| 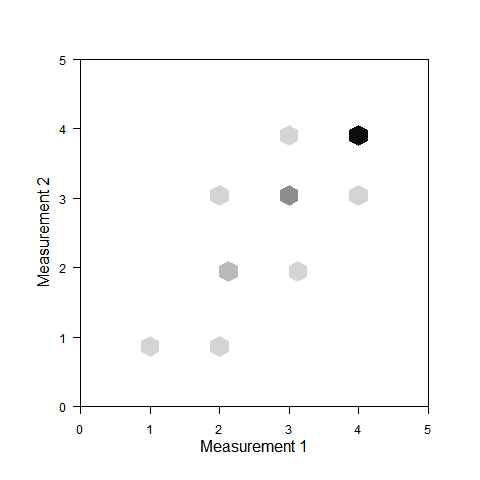 | 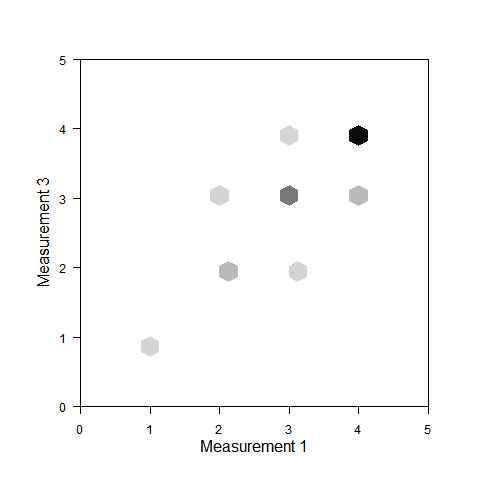 | 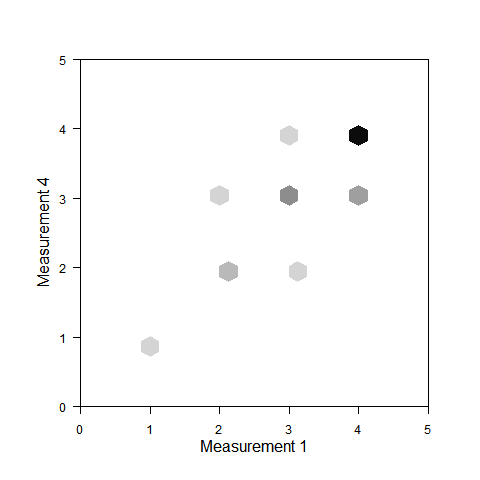 |
| --- | --- | --- |
| 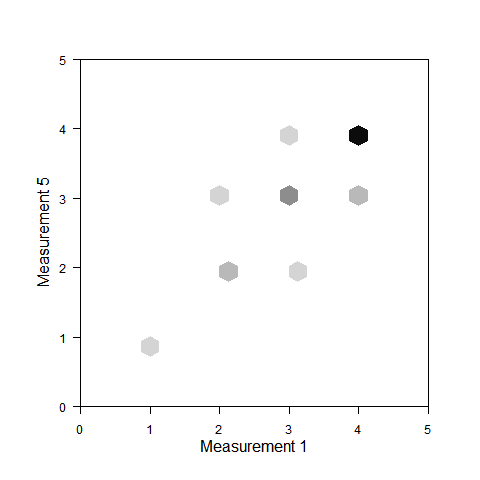 | 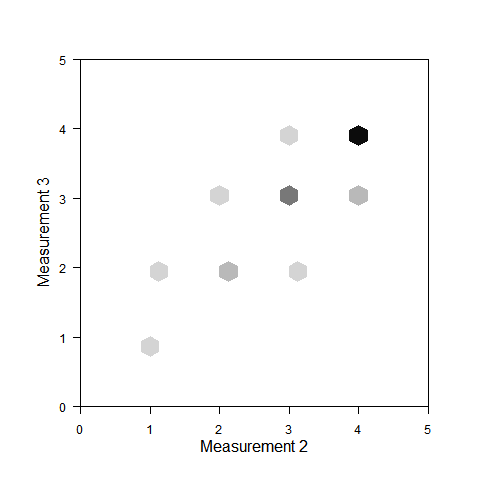 | 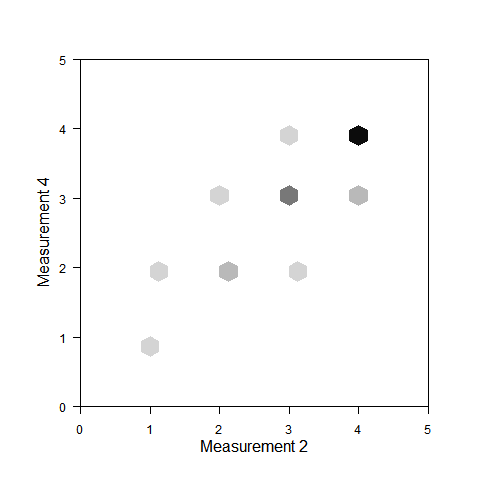 |
| 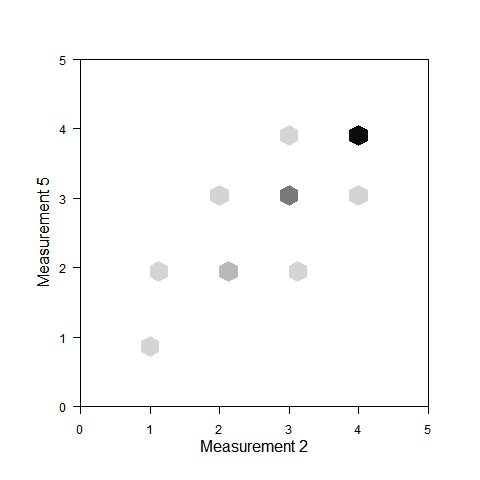 | 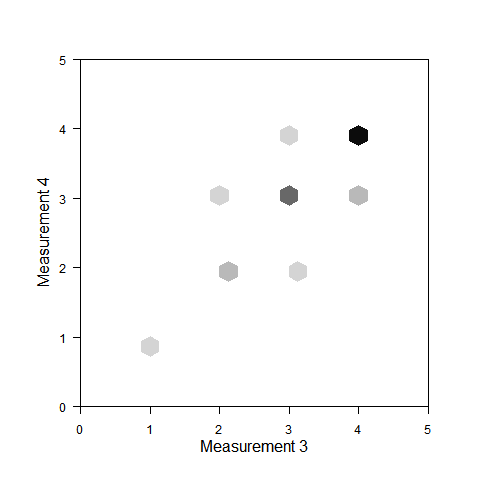 | 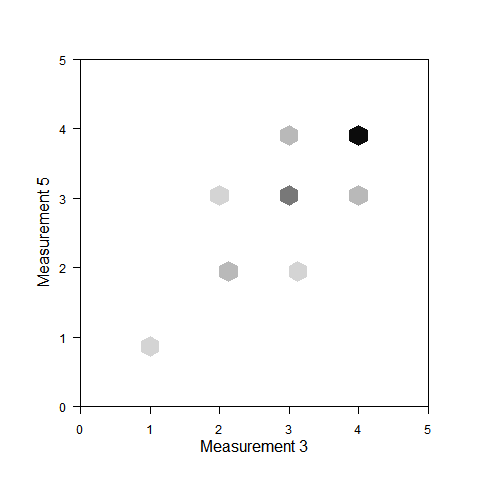 |
| 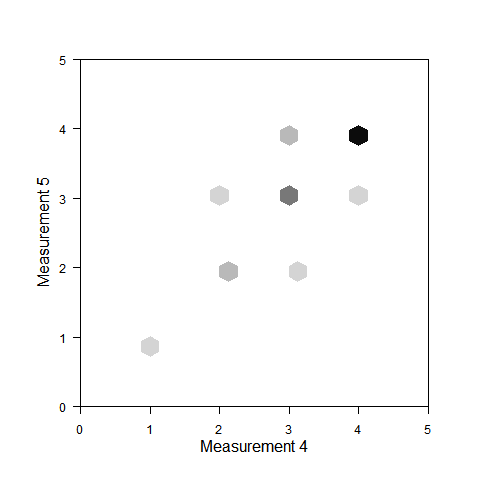 |  |  |

Supplement 7: Intra-observer reliability – Plumage measures possible repeated measurment pairs. Darker points represent more overlapping scorings.

|  |  |  |
| --- | --- | --- |
|  |  |  |
|  |  |  |
|  |  |  |

Supplement 8: Intra-observer reliability – Fat score correlation all possible repeated measurment pairs.

Table S1: Theoretical Scoring example of two observers 1 and 2 for two scorings scenarios A and B to illustrate the problem of the summed-up plumage score in the reliability analysis with respect to otherwise hidden disagreements. This illustrated our approach for the reliability analysis, we only for the validity did not calculate total plumage score, i.e., the sum of all six plumage scores, but the six single scores of all six body parts were used, as this is a more conservative approach. This ensures that one can see exactly where the observers have scored different values, and subtle disagreements are not summed out. In the below shown theoretical example, the scoring scenarios A and B show a good agreement if one considers only the calculated sum of the plumage scores. But if we take a look at the single scores of the six different body areas, this impression changes. In scenario A, the difference in scoring points is only one. In scenario B, the difference in scoring points is 10. Both plumage scores seem to show a good agreement, but only scenario A indeed agrees well.

| Alternative scenario |  | head | neck | back | tail | wing | throat | Plumage score |
| --- | --- | --- | --- | --- | --- | --- | --- | --- |
| A | Observer 1 | 2 | 4 | 3 | 4 | 4 | 2 | 19 |
|  | Observer 2 | 2 | **3** | 3 | 4 | 4 | 2 | **18** |
| B | Observer 1 | 4 | 3 | 4 | 4 | 2 | 3 | 19 |
|  | Observer 2 | **1** | **4** | **3** | **2** | **4** | **4** | **18** |
